# STEPS: efficient simulation of stochastic reaction–diffusion models in realistic morphologies

**DOI:** 10.1186/1752-0509-6-36

**Published:** 2012-05-10

**Authors:** Iain Hepburn, Weiliang Chen, Stefan Wils, Erik De Schutter

**Affiliations:** 1 Theoretical Neurobiology, University of Antwerp, Campus Drie Eiken, Universiteitsplein 1, Wilrijk,2610, Belgium; 2 Computational Neuroscience Unit, Okinawa Institute of Science and Technology, Seaside House, 7542, Onna, Onna-son, Kunigami, Okinawa 904-0411, Japan

## Abstract

****Background**:**

Models of cellular molecular systems are built from components such as biochemical reactions (including interactions between ligands and membrane-bound proteins), conformational changes and active and passive transport. A discrete, stochastic description of the kinetics is often essential to capture the behavior of the system accurately. Where spatial effects play a prominent role the complex morphology of cells may have to be represented, along with aspects such as chemical localization and diffusion. This high level of detail makes efficiency a particularly important consideration for software that is designed to simulate such systems.

****Results**:**

We describe STEPS, a stochastic reaction–diffusion simulator developed with an emphasis on simulating biochemical signaling pathways accurately and efficiently. STEPS supports all the above-mentioned features, and well-validated support for SBML allows many existing biochemical models to be imported reliably. Complex boundaries can be represented accurately in externally generated 3D tetrahedral meshes imported by STEPS. The powerful Python interface facilitates model construction and simulation control. STEPS implements the composition and rejection method, a variation of the Gillespie SSA, supporting diffusion between tetrahedral elements within an efficient search and update engine. Additional support for well-mixed conditions and for deterministic model solution is implemented. Solver accuracy is confirmed with an original and extensive validation set consisting of isolated reaction, diffusion and reaction–diffusion systems. Accuracy imposes upper and lower limits on tetrahedron sizes, which are described in detail. By comparing to Smoldyn, we show how the voxel-based approach in STEPS is often faster than particle-based methods, with increasing advantage in larger systems, and by comparing to MesoRD we show the efficiency of the STEPS implementation.

**Conclusion:**

STEPS simulates models of cellular reaction–diffusion systems with complex boundaries with high accuracy and high performance in C/C++, controlled by a powerful and user-friendly Python interface. STEPS is free for use and is available at http://steps.sourceforge.net/

## **Background**

As the understanding of the molecular systems governing many aspects of cellular function improves it is becoming increasingly clear that the assumption of mass action kinetics in well-mixed volumes is often invalid. A good example is calcium signaling, which can be highly localized with very steep concentration gradients [1-3]. Calcium signaling depends on the interaction between membranes where the calcium channels are located and the cytoplasm where calcium activates many different enzymes [2,3]. The membrane channels are often arranged into clusters containing only a few or tens of channels [4,5] resulting in stochastic release events that have been observed experimentally [6]. In neurons, the resting concentration of calcium in dendritic spines, where it plays an essential role in triggering synaptic plasticity, corresponds to only a few ions in this small volume [7], indicating that calcium dynamics can be highly stochastic [8]. Moreover, dendritic spines have a typical morphology that strongly affects the inward and outward diffusion of molecules [9]. Taken together, these considerations point to the need of software that supports the simulation of stochastic reaction–diffusion systems with an accurate representation of the complex geometries specified by the membranes of a cell and its intracellular organelles. In this paper we describe STEPS, STochastic Engine for Pathway Simulation, which was designed to give modelers an efficient implementation with a sophisticated user interface.

Stochastic reaction–diffusion can be solved using two fundamentally different approaches: particle-based or voxel-based methods. In the first method one keeps track of the Brownian motion of each individual molecule in the simulation and reactions are based on collisions between molecules, while in the second approach the behavior of groups of molecules in subvolumes is computed using the laws of chemical kinetics, and diffusion is simulated as the transport of molecules from one subvolume to another. Particle-based methods can be further divided into those that track Brownian motion in open space (examples are MCell [10,11] and Smoldyn [12]) or those that use lattices on which molecules hop from one site to another (examples are GridCell [13] and see [14,15]). An advantage of these methods is the high physicochemical fidelity of the approach, but this comes at the price of having to track the behavior of every single molecule in the system. This is computationally expensive and may not always be relevant in a biological context.

Stochastic voxel-based approaches compute changes in the number of molecules present in small volumes without distinguishing among individual molecules. This can be more efficient in large systems and also allows for easier combination of exact solution methods with approximative ones [16], which may greatly speed up computations (see Discussion for more detail). A widely used approach to model chemical reactions is Gillespie’s Stochastic Simulation Algorithm (SSA) [17], which can easily be extended to deal with diffusion (see further and [17,18]), a method commonly referred to as “spatial SSA” or “spatial Gillespie”. STEPS implements a derivative of the SSA in tetrahedral meshes to model the geometry, which importantly allow for a much better morphological resolution than the cubic voxels used in most other SSA based software, e.g. MesoRD [19] and NeuroRD [20].

This paper describes STEPS 1.3 and is structured as follows: we first introduce the overall workflow and structure of STEPS and its multiple solvers, the rest of the paper largely focusing on the solver for stochastic reaction–diffusion. We next introduce the SSA, tetrahedral meshes and how to adapt the SSA to model diffusion in such meshes. We then demonstrate the accuracy of STEPS and compare efficiency to other reaction–diffusion simulators. We finish with describing the Systems Biology Markup Language (SBML) [21] import module and demonstrate simulation of some SBML models using STEPS.

## **Implementation**

### **STEPS overview**

**Figure 1:**
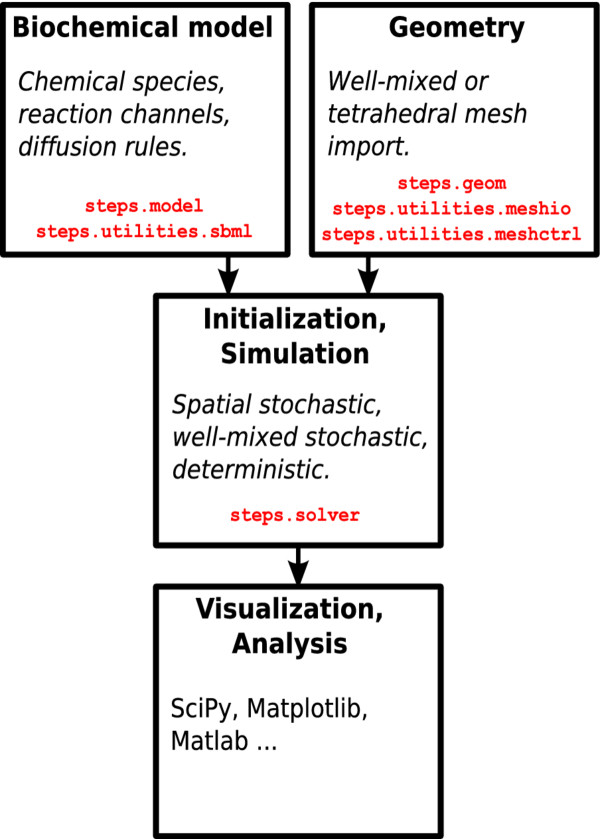
**STEPS workflow. **The biochemical model and the geometry are described separately (using Python modules steps.model and steps.geom respectively) and are brought together by the solver object. The steps.utilities namespace contains various helper modules that assist in model and geometry construction. Python packages such as SciPy are a convenient tool for post-simulation analysis.

The user interface to STEPS is in Python, a very powerful and versatile scripting language, while the core STEPS code is in C/C++ for high efficiency. Figure 1 shows a typical STEPS workflow. Everything in the Python user front-end is contained in namespace ‘steps’, within which there are a number of modules that contain classes and functions separated by the different tasks required to build a STEPS simulation. This means that using STEPS largely consists of creating Python objects to represent the various components of a reaction–diffusion model (e.g. chemical species, reaction and diffusion rules, compartments etc.) and invoking their methods to set conditions and to control the simulation.

STEPS differs from many reaction–diffusion simulators in that the chemical model and the geometry are described completely separately, which can be an advantage due to the challenging task of creating a suitable complex geometry for spatial simulations. For example, with the two tasks separated in this way one researcher may dedicate all their time purely to mesh-construction while another researcher constructs the biochemical model. Meshes may then be saved, shared and reused in other simulations as required. Uncoupling the model description from the simulation means that complex initial conditions can be achieved [22] and modified without making changes to the model. To compliment these possibilities compatibility with current developed standards such as SBML is also achievable, yet relying purely on SBML would disable some important features of STEPS such as those mentioned above.

Running a STEPS simulation will usually involve creating one main Python module, which will import STEPS modules and possibly other outside user-written modules for the model (such as separate modules for tetrahedral mesh description or user-defined helper functions) along with some of the many powerful scientific tools available for Python such as SciPy and NumPy. Python is generally regarded as a relatively easy-to-learn, intuitive language and the basic skills required to run a STEPS simulation - such as creating and manipulating objects, running simple loops and perhaps reading and writing to files - can usually be acquired quickly. We give a brief overview of the main components of the STEPS Python user interface:

#### steps.model

The steps.model module contains everything required to describe how chemical species in the model interact. For example, the chemical species themselves are described by creating instances of class steps.model.Spec. Interactions of chemical species are described by creating objects to represent chemical reactions and diffusion rules. At this stage nothing is said about where these interactions take place, although different rules are grouped into ‘volume systems’ and ‘surface systems’, which are the objects that connect the biochemical model with the geometry description.

#### steps.geom

The steps.geom module contains all the classes and functions required to describe geometry to which a biochemical model may be applied. The basic building blocks of geometry in STEPS are ‘compartments’ and ‘patches’. A compartment is a 3D volume with reflective boundaries in which molecules may diffuse and react, and can either be well-mixed (therefore defined only by volume), or described by a collection of tetrahedrons in a mesh. A patch is a 2D surface in which molecules may be embedded and is connected to one or two compartments. Analogously to compartments, in a well-mixed description patches are defined only by area and in a spatial simulation they are described by a collection of triangles forming a surface within a tetrahedral mesh. ‘Surface reactions’ may take place in patches, which describe both surface-volume and surface-surface reactions allowing, for example, a molecule that is diffusing in a volume to become embedded in a surface and a molecule that is embedded in a surface to diffuse to a neighboring volume. Such features are used to model events such as ligand-binding and transport.

Groups of reaction and diffusion rules (‘volume systems’) defined in the biochemical model may be added to all (or a selection of) compartments in the geometry, and any groups of defined surface reaction rules (‘surface systems’) may similarly be added to patches. Grouping in this way can bring advantages of realism and complexity, such as allowing the mobility of a species to differ between different environments, and performance because, for example, it is possible to declare which reactions from the overall set of reactions will occur in any given compartment and thus save memory by omitting reactions that can never occur (if, for example, a reactant species never appears in that compartment).

The overall geometry used for any given simulation must be either a collection of well-mixed compartments and patches, or a collection of compartments and patches all within a tetrahedral mesh. The steps.geom.Tetmesh class, which represents a tetrahedral mesh, contains a vast amount of information about the tetrahedrons and the triangle surfaces in the mesh and their connectivity with many helper functions for retrieving this information. This can be vital for initializing conditions in and running a spatial simulation.

#### steps.rng

The steps.rng module contains the “Mersenne Twister” [23] random number generator class that provides the random numbers required by the STEPS algorithms.

#### steps.solver

The steps.solver module contains all the simulation solvers available in STEPS. A solver requires access to a biochemical model description along with a geometry description in order to build and run a simulation. All solver classes are derived from an abstract base class, which means all solvers contain some shared functionality such as the ability to inject molecule species into a compartment, run a simulation for some time and record updated concentrations. Separate solvers then implement some or all of the optional methods depending on whether the function makes sense for that particular solver. The main focus for this work is solver ‘Tetexact’, which is a stochastic solver that supports complex morphology and diffusion and is described further in Implementation of spatial SSA solver. In addition there are two other solvers available in STEPS 1.3: ‘Wmdirect’, which is a well-mixed stochastic solver based on Gillespie’s SSA [17], and ‘Wmrk4’, which is a deterministic solver based on the Runge–Kutta method [24].

### **Gillespie stochastic simulation algorithm (SSA)**

A brief description of the direct method formulation of the SSA and its implementation in the Wmdirect solver can be found in the Additional file 1. For a more detailed overview of the algorithm and its background see [16,25-27]. The SSA is an event-driven algorithm that has been demonstrated to give an exact solution to the chemical master equation [17]. The fundamental assumption made of the system is that elastic (non-reactive) collisions greatly outnumber reactive ones, which means that molecules become distributed uniformly throughout the system volume and that their velocities become thermally randomized to the Maxwell-Boltzman distribution. Further, molecules are assumed to occupy a volume which is negligible in comparison to the total system volume.

Several optimizations to the original direct method exist [25], such as the construction of a dependency graph [28] so that only the propensities of affected reaction and diffusion channels are updated in each iteration. Such an approach is adopted by STEPS in solvers Wmdirect and Tetexact, as well as some other subvolume-based software (e.g. MesoRD).

### **Implementation of spatial SSA solver**

As mentioned, the standard SSA assumes a well-mixed system. However, one can introduce spatial gradients into the SSA [17] by modeling a system of well-mixed subvolumes with diffusion between them described as first-order reactions.

In the well-mixed formulation the reaction container is described only by its volume. In the spatial SSA solver this reaction container is broken up in *N*_*tet*_ smaller subvolumes and each of these subvolumes is treated as a reaction container in its own right by cloning the reaction channels. This means that for *M* reaction rules, and assuming all reactions may occur in all tetrahedrons (which is not required by STEPS - see STEPS overview), the total number of reaction channels will be *M*_*reac*_ = *N*_*tet*_ * *M*. The state **x** of the simulation will also become much bigger, it consists of *N* * *N*_*tet*_ integers, with **x**_i,k,t_ representing the number of molecules of species *i* in subvolume *k* at time *t*.

If we make the subvolumes within a certain size window (see Subvolume size), the well-mixed assumption applies to each subvolume independently and one can accurately represent concentration gradients [29]. The rate of diffusion of molecules of species *i* with diffusion constant *D*_*i*_ between two neighboring subvolumes will depend on the shape of the subvolumes. In STEPS we have chosen to use tetrahedral meshes, a type of non-orthogonal, unstructured mesh in which the problem domain is decomposed into a connected set of tetrahedral elements [30]. Since the tetrahedra do not have to be perfectly regular, they can smoothly follow any boundaries and can adapt their size to the local level of detail. Quite often the subvolumes in these meshes are the Voronoi elements surrounding all edge nodes. STEPS instead uses the tetrahedrons themselves, meaning that each tetrahedral voxel has 4 triangular sides and, through them, is connected to a maximum of 4 neighboring tetrahedra. Compared to the Voronoi description this reduces coding complexity (Voronoi elements have various numbers of neighbors), maintains control over subvolume size and allows for a far more accurate description of a surface that represents a membrane within a mesh.

Diffusion of chemical species *A* between neighboring tetrahedrons *k* and *l* is simulated by the following reversible “reaction” channel:

(1) $$A_{k}\overset{d_{k,l}}{\underset{d_{l,k}}{⇌}}A_{l}$$

With the diffusion rates given by:

(2) $$\begin{array}{l} d_{k,l}=\frac{D_{i}S_{i,k}}{V_{k}dx_{k,l}}\\ d_{l,k}=\frac{D_{i}S_{i,l}}{V_{l}dx_{l,k}} \end{array}$$

Where *S* is the surface area of the triangle connecting tetrahedrons *k* and *l*, *V* is the volume of the tetrahedron and the distance *dx* is computed as the barycenter-to-barycenter distance, therefore *dx*_*l,k*_ == *dx*_*k,l*_.

Including diffusion greatly increases the total number of reactions channels: *M*_*tot*_ = *M*_*reac*_ + *M*_*diff*_, with *M*_*diff*_ < 4 * *N* * *N*_*tet*_ (internal tetrahedrons are connected to 4 neighbors, but tetrahedrons at boundaries are connected to fewer). However, a diffusion channel in STEPS actually consists of diffusion of a particular species from a tetrahedron to any one of its neighbors, with a total rate of diffusion equal to the sum of the rates in each direction. Once a diffusion channel is chosen by the SSA, one of the possible directions is then chosen. This is mathematically equivalent to describing diffusion of a particular species from a tetrahedron as 4 (maximum) separate diffusion channels, but reduces memory requirements by approximately a factor of 4 with only a small cost to efficiency.

The use of a tetrahedral mesh greatly increases the complexity of the search for the next reaction in the SSA. Since version 1.3, the implementation of Tetexact solver has therefore adopted the Composition and Rejection (CR) Method [31], whose time complexity is constant even for systems with large number of reactions. The algorithm is described in more detail in Additional file 1.

### **Importing and annotating tetrahedral meshes**

High quality meshes are often essential to obtain accurate simulation results. Instead of developing our own mesh generator we make use of powerful mesh generation software, such as CUBIT [32], TetGen [33], and Gmsh [34], and provide a set of utilities for importing tetrahedral meshes. This approach is significantly different from the approach taken by most other spatial SSA simulators, for example MesoRD [19] and NeuroRD [20], where cubic mesh generation is included in the simulation.

The mesh importing utilities are carefully designed so that STEPS is not only capable of importing meshes from supported formats, but is also extendable. Currently, one-step importing functions support three common mesh formats: the Abaqus format exportable by CUBIT, TetGen’s own formats (.node, .ele and .face), and the MSH ASCII format used by Gmsh. These import functions use the pure-Python-based ElementProxy class which provides generic mesh importing functionalities such as data storage, grouping and index mapping. During import, first a sufficient number of ElementProxy objects are created of a type corresponding to the kind of geometry element in the mesh data (e.g. tet_proxy for tetrahedrons). After that, data about each geometry element is inserted in its associated proxy. During the insertion, the proxy automatically assigns a STEPS index for the element and also records the index mapping between the import index and STEPS internal index of the element, which are accessible during later simulation. Once all element data is inserted, the proxy objects can be directly used to create meshes, compartments and patches in STEPS, as well as to perform further mesh manipulations.

**Figure 2:**
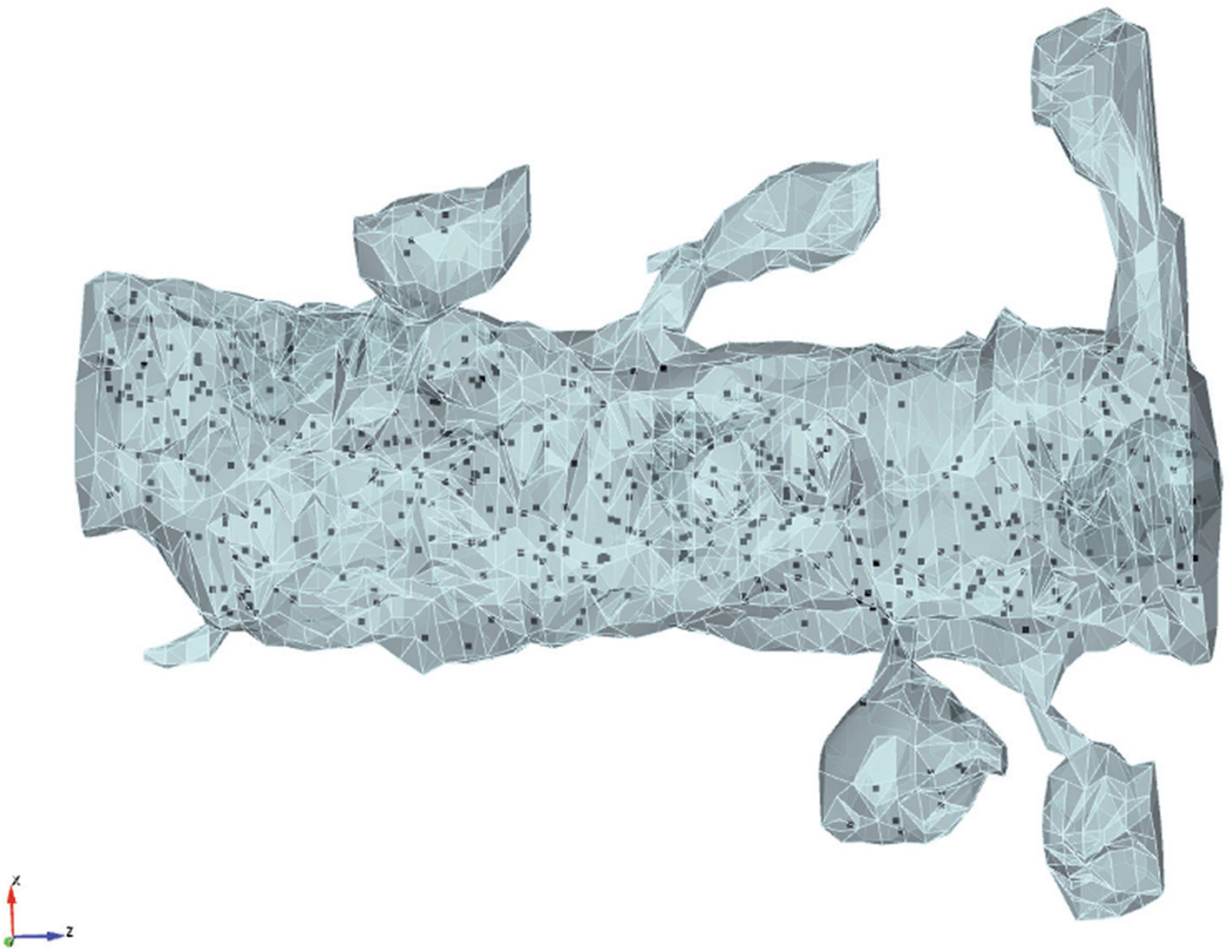
**STEPS import of a tetrahedral mesh with realistic geometry. **The mesh is reconstructed from a surface triangular mesh provided at http://synapses.clm.utexas.edu/anatomy/Ca1pyrmd/radiatum/K24/K24.stm . For a test simulation in STEPS molecules are distributed uniformly and the system is then visualized in CUBIT.

The mesh importing utilities in STEPS also provide a more advanced, flexible way to simulate systems with complex geometries. Traditionally, meshes for subvolume-based SSA simulations have been constructed from combinations of standard geometry primitives such as cubes, spheres and cylinders [9], where geometry features are highly abstracted. A typical representative of this method is the Constructive Solid Geometry (CSG), adopted by MesoRD for geometry construction. This type of mesh is relatively easy to construct, but the highly abstracted models may not reflect real geometry constraints to the system and may produce inaccurate simulation results (see Results). A better, yet more challenging approach is to reconstruct volume meshes from biological data based on closed surface meshes. However, the surface meshes derived from series of electron microscope images [35] are commonly unclosed and have small intersecting surfaces, thus they cannot be used directly in volume mesh generation. This problem can be solved by semi-manually preprocessing the surface meshes using mesh manipulation tools such as MeshLab [36]. Once volume meshes are generated from the cleaned-up surface meshes, they can be imported to STEPS for simulations. Figure 2 gives an example STEPS simulation running in a reconstructed mesh with realistic geometry.

#### ***Subvolume size***

When generating a mesh for STEPS, and other stochastic voxel-based approaches to reaction–diffusion simulation, an important consideration is the size of the subvolumes. As described in Implementation of spatial SSA solver, subvolumes are assumed to be of a size that represents a well-mixed region. Most real biological systems will indeed exhibit a size-band where the natural motion of molecules maintains the well-mixed condition and for a STEPS simulation we should ensure that all tetrahedrons fall within this band for maximum simulation accuracy.

How can we estimate the size at which a system exhibits well-mixed behavior? By ‘well-mixed’ (or ‘well-stirred’) we mean that there are many more nonreactive collisions than reactive ones, quickly removing any spatial gradients that appear from phenomena such as chemical reactions or transport. At relatively large volumes, however, spatial chemical gradients can persist in a region, broadly speaking whenever reactions occur at a faster rate than the region can be smoothed by diffusion. If we were to represent such regions with well-mixed subvolumes we would lose spatial detail. Determining the largest volume at which spatial gradients don’t exist is a good estimate of the upper-bound for the well-mixed condition and can be estimated mathematically by comparing reaction time to diffusion time in the continuous case. However, it would be naive to assume that below a certain volume the region is always well-mixed; subvolumes can also be too small [37,38]. Sizes that are comparable to the size of a molecule are intuitively too small since we require that molecules are well-defined within subvolumes, and sizes smaller than the mean-free path of a molecule are also too small because there may not be enough elastic collisions taking place inside a region to keep it well-mixed. In a discrete description the minimum number of molecules in a *populated* tetrahedron is 1, which means that, below a certain size (where the mean number of molecules per tetrahedron becomes much less than 1), reaction time for a *populated* tetrahedron *decreases* with decreasing volume (reaction *rate* increases) and comparison to diffusion time for the discrete case is a good way to estimate the minimum subvolume size.

So there exists a window of subvolume size at which we can apply the well-mixed approximation, which may be slightly different for each particular model. It is important to determine this window, but we will show that it is usually relatively large for biologically realistic models and therefore poses only a minimal restriction on mesh-generation. Simulation time increases with increasing number of tetrahedrons, so tetrahedron size is also an important consideration for simulation efficiency. The greatest simulation efficiency possible with acceptable accuracy would be with all tetrahedrons in the mesh at the upper-bound of acceptable size. However, the tetrahedrons that represent the space around complex boundaries may have to be significantly smaller than the largest acceptable size so as to represent the boundaries accurately. An ideal mesh for any given problem, therefore, is one that achieves the greatest simulation efficiency at which the well-mixed assumption holds, but with acceptable morphological resolution.

For an example problem with reasonable simulation parameters, if the fastest reaction in the system is a second order reaction $A+B\overset{k}{\to }C$ with k = 100/μM.s, the slowest diffusion coefficient is D = 0.1 μm^2^/ms, and concentrations are [A] = [B] = 1 μM, our upper bound estimate is approximately 0.4 μm with a lower bound of approximately 0.02 μm (see Additional file 2). This size window is large enough not to place much restriction on our mesh-generation at all. Though a factor of 20 for the tetrahedron *size* may not sound like a large window, it means of the order of a 10^4^ factor difference in *volume*. Put another way, the number of tetrahedrons per cubic micron of mesh for this problem should number more than approximately 100 and fewer than approximately 1,000,000 to ensure the well-mixed subvolume condition. There are several reasons why we may wish to stay significantly larger than the calculated lower bound in this example, the practical reasons that a mesh of 1 million tetrahedrons per cubic micron would consume enormous amounts of memory and result in an unnecessarily slow simulation, and another reason is that at 20 nanometers we are approaching the size of proteins.

**Figure 3:**
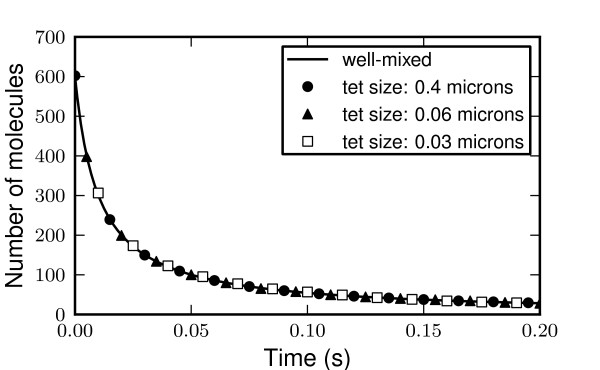
**Subvolume size. **The model described in the text is simulated for different uniform meshes over a range of tetrahedron sizes, all within the acceptable upper and lower bound of 0.4 μm to 0.02 μm, and each mesh representing the same geometry. Mean results for 10 iterations in each case are identical for each mesh size with no errors, demonstrating the accuracy of the well-mixed approximation in this range.

Figure 3 shows the simulation of this system in STEPS with three different tetrahedral meshes representing the same total volume of one cubic micron. The three meshes are reasonably regular and range from the upper bound of accepted subvolume size (with approximately 100 tetrahedrons) to a size of 30 nanometers (approximately 350,000 tetrahedrons). There are no significant errors in results and no discrepancies between the different mesh sizes, showing that the spatial Gillespie method is accurate over this range for this simple problem.

We may find for other types of simulations that our window may even be larger than in this example. However, with slower diffusion the window may become narrower, but is often still large enough not to pose too much restriction on mesh generation. For example, with the same example parameters except for a diffusion coefficient of 0.02 μm^2^/ms, which is about the slowest diffusion coefficient of the very largest proteins in water [39-41], the mesh should contain more than approximately 300 tetrahedrons and fewer than approximately 20,000 tetrahedrons per cubic micron, so some care should be taken in this case not to go below the lower bound of acceptable subvolume size (i.e. not going *higher* than 20,000 tetrahedrons). The crowded environment of the cell can cause the observed apparent diffusion coefficient to be lower than that in water by a factor that may be dependent on the size of the molecule [41]. However, crowding at the same time is expected to also *decrease* the rate of fast, diffusion-limited association reactions [42-44]. Therefore a scenario where large, very slowly-diffusing molecules are involved in fast reactions that increase the minimum subvolume size enough to approach the upper bound is rather unlikely. The Results section contains a validation of the well-mixed subvolume calculation for a problem with slow diffusion, approximately in the range for the apparent diffusion coefficient measured of large proteins in the cytoplasm.

Recent proposals have been made to correct reaction rates at the algorithmic level if subvolumes approach a small, “critical” size [38], however in STEPS we prefer to keep the larger *minimum* size as a constraint on the model, in effect constraining the subvolume size to be significantly larger than the “critical” size. This is partly because the small sizes typically involved often consume huge amounts of memory and slow simulations unnecessarily or may even be unattainable, so to go below the lower bound and approach the critical size is often impossible or impractical. In the case for very slow diffusion it may be necessary for modelers to take some care to ensure tetrahedrons do not become too small, but in practice, for most biologically realistic models, mesh generation is not significantly restricted by the subvolume size consideration and the major challenge is realistic boundary representation.

#### ***Mesh quality***

Consideration should also be given to the quality of the mesh used for the STEPS simulation. Tetrahedrons, compared to cubic elements, have the benefit of being able to adapt their size and shape to local levels of detail, however one must make sure that they do not become too irregular, or “stretched” in any regions of the mesh. In part this is due to considerations of subvolume size (if a tetrahedron is stretched the size of the tetrahedron in some directions may be much larger than the edge-length assumed for the regular tetrahedron) and also to do with an assumed level of regularity for the derivation of the diffusion rates.

There are many different quality measurements for a tetrahedron and each mesh-generator usually comes with one or more from this set. The software may use these measures internally so as to ensure good quality mesh output, and can also report quality of the generated mesh to the user. The quality measure used in TetGen is the radius-edge ratio: the ratio of the radius of the tetrahedron’s circumsphere to the length of the shortest edge. This value is approximately 0.61 for a perfectly regular tetrahedron. Values up to 2.0, which is currently the default value in Tetgen for Quality mesh generation, produce a mesh in which no tetrahedron is too stretched out. CUBIT incorporates many quality measurements, for example the Aspect Ratio Beta [45], which is the circumsphere radius divided by 3 times the inscribed sphere radius and takes acceptable values between 1 (regular tetrahedron) and 3.

STEPS itself comes with a quality measure that can be performed on the imported mesh, the radius-edge ratio that is also used in TetGen. As well as quality, it may be desirable for example to find the minimum, maximum and standard deviation of tetrahedron volumes so as to ensure they fall within the acceptable range, and this can be achieved with a simple loop over mesh elements in the Python interface. STEPS will not fail to run a simulation on a poor quality mesh, since setting an internal tolerance may be too restrictive, so users should decide whether to perform their own analysis on a mesh to determine if it is of acceptable quality before running a simulation.

## **Results**

### **Accuracy of geometry representation**

To test our intuition that complex geometry may be better represented by tetrahedral meshes than cubic meshes we constructed five geometrical shapes each representing a dendritic spine on a neuron [7,9] by a simple combination of a spherical head and cylindrical neck. All spines were generated randomly within constraints to cover a broad range recorded experimentally from rat Purkinje neurons [46] (see Additional file 3). We then compared the accuracy of these meshes for two biologically important measures: volume, important for chemical reactions and diffusion in the spine, and surface area, important for membrane transport mechanisms like voltage-gated calcium channels on the spine [1].

**Figure 4:**
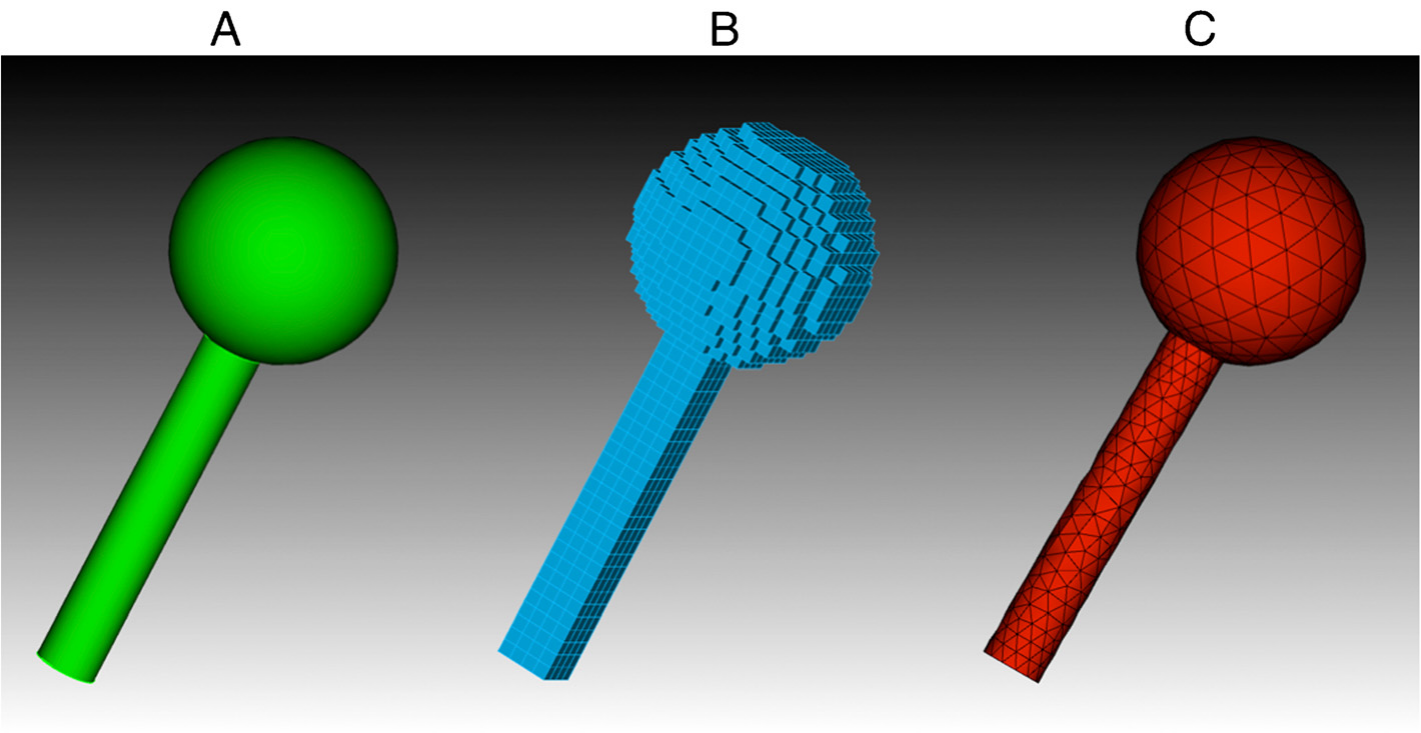
**Example tetrahedral and cubic spine meshes. **The ideal geometry for Spine #4 (**A**) represented by a cubic mesh of 2576 cubes (**B**) and by a tetrahedral mesh of 2571 tetrahedrons (**C**). The mesh surfaces are displayed in CUBIT.

For each spine shape, first an adaptive tetrahedral mesh was generated in CUBIT with the coarsest mesh (minimal number of tetrahedrons) permissible by the software, and then a cubic mesh was generated in MesoRD from CSG input, with the cube size controlled to result in a mesh with a similar number of subvolumes to the tetrahedral mesh (further information about the meshes can be found in Additional file 3). Furthermore, for each spine a more detailed (greater number of subvolumes) tetrahedral and cubic mesh was generated, with a close match between the number of tetrahedral and cubic subvolumes. The more detailed meshes typically approached the approximate minimum subvolume size for a system of slow diffusion and fast reaction previously discussed, and so are approximately the most detailed mesh that would be acceptable for simulation. Figure 4 shows spine #4 represented by both a tetrahedral mesh and cubic mesh in the coarser case.

**Figure 5:**
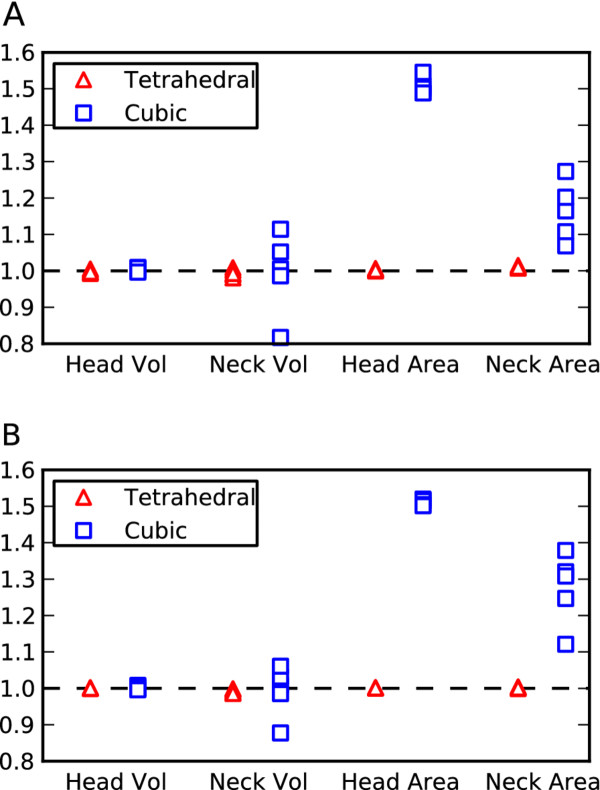
**Comparison between cubic and tetrahedral spine meshes. **A scatter plot of the properties of the tetrahedral meshes (red triangles; many points overlap) and the cubic meshes (blue squares; some overlap) representing geometry which consists of a spherical head joined to a cylindrical neck to approximate dendritic spines. All properties were plotted as a ratio of measured value/ideal value. **A**. The coarsest meshes ranging from approximately 2500–3000 subvolumes per mesh. **B**. The more detailed meshes ranging from approximately 11000 to 16000 subvolumes per mesh.

For all meshes the volume and surface area of both the head and neck regions were measured and compared. Figure 5 shows a plot of the normalized measurements. All meshes appeared to represent the spine head volume quite accurately, yet the cubic meshes often failed to represent the neck volume sufficiently, and only a marginal improvement was noticeable in the more detailed meshes. This demonstrates that, while one could always find an optimal cube size to represent any one region of a geometry accurately, the cube size will not necessarily suffice for other regions which may have different morphologies. This is a clear drawback for cubic meshes, which originates from the need for all subvolumes to be of the same size. Any error in volume will of course produce an error in reaction rates as well as for diffusion rates. It may be possible with a very detailed mesh to represent all regions sufficiently, yet a larger number of subvolumes means a slower simulation and may result in loss of accuracy caused by the small subvolume size.

In terms of surface area in all cases the cubic meshes failed to represent the boundaries accurately, in fact slightly worsening in the more detailed meshes. This obviously arises from a discrepancy between the surface/volume ratio of a cube compared to a sphere or cylinder. This failure to represent surface area closely could for example be important if modeling the mobility of surface molecules, or if a density of surface molecules is specified in a model then the total number of molecules would end up being too high in a cubic mesh due to the larger surface area. In such a case it may be possible to overcome such difficulties by introducing a correction factor, yet a further complication is that, in tests, we determined that such a correction factor is not constant and varies considerably throughout regions of the spine meshes.

In all cases the tetrahedral meshes represented volume and surface area throughout all regions of the mesh with high accuracy.

### **Validation**

Although STEPS uses established methods to simulate reaction–diffusion systems, errors can be made in the coding that would lead to erroneous results. Therefore it is important to validate the accuracy of the program by simulating models for which the correct response is known. To our knowledge no standard benchmarking library for reaction–diffusion systems exists, so we developed a representative set of models that test different aspects of the code.

Here we briefly describe each model and show the accuracy of the results in STEPS. The models and parameters used are described in detail in Additional file 4 and in the model scripts which can be downloaded from the STEPS website.

#### ***Simple well-mixed reactions***

We tested STEPS accuracy for four types of reactions, chosen for their prevalence in real systems and models, as well as for their fitness for analytical investigation. The Python interface to STEPS made it easy to take the mean and standard deviation of a large number of individual simulation runs and in each case the mean STEPS output was converted to a 95% confidence interval (CI), which was then compared to the known analytical value (detailed in Additional file 4).

**Figure 6:**
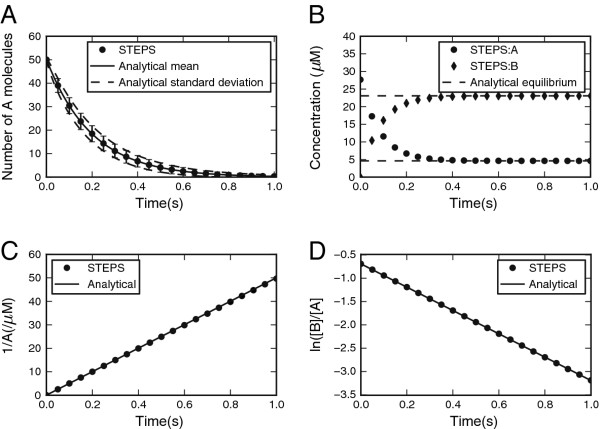
**Validation of reactions. A. **First-order irreversible reaction. Mean for 1000 iterations of STEPS (points with error bars showing the sd) matches the analytical solution (full line, broken lines are the predicted sd). **B.** First-order reversible reaction. The STEPS simulation (mean of 100 iterations) evolves correctly to the predicted steady state concentrations. **C, D.** Second order irreversible reaction. With equal reactant concentrations the evolution in time of the inverse of the concentration of one of the source species in comparison to the analytical solution is shown (C) and with unequal reactant concentrations the comparison of the source species ratio with the analytical solution is shown (D).

One of our simplest validation systems, the first-order irreversible reactions system, is also perhaps one of our most important due to the fact that we test the resulting noise from our implementation of the SSA. This vital aspect of stochastic reaction–diffusion simulator output is usually insufficiently tested, often with simple visual comparison of the amplitude of the noise from the output of two different simulators. The standard deviation matched the analytical solution to the chemical master equation closely, and the mean behavior also behaved as expected (20 of 20 points fell in the 95% CI) (Figure 6A).

For first-order reversible reactions the steady state can be computed and the mean concentrations of the STEPS simulation evolved properly to this steady state (14 of 14 points in CI) (Figure 6B). For the second-order irreversible reaction with equal reactant concentrations (Figure 6C) and unequal concentrations (Figure 6D) the mean behavior of the STEPS simulations followed the analytical solutions (38 of 40 and 19 of 20 points within CI respectively).

**Figure 7:**
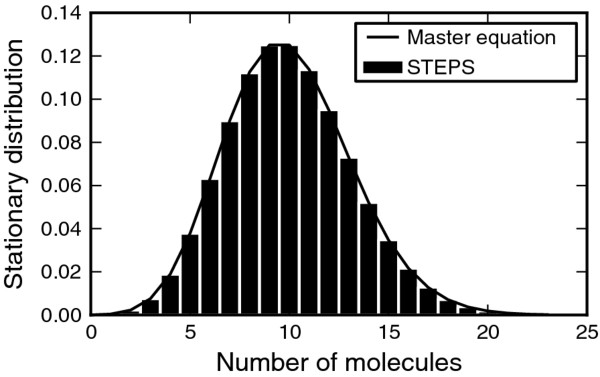
**Validation of production and degradation reactions. **Compares the stationary distribution of a combined production and degradation reaction of a single species from the STEPS simulation (histogram) with the analytical solution (solid line).

We also tested a ‘Production and Degradation’ reaction system described by two reactions: a first-order annihilation reaction and a zero-order production reaction. Such reactions, though they may be unphysical in biological systems, are useful simplifications that are commonly used in models and as such are supported in STEPS. Due to the simplicity of this system an analytical solution to the steady-state version of the chemical master equation can be found (see Additional file 4). The stationary distribution from the simulation in STEPS followed the analytical prediction (15 of 16 points within CI) (Figure 7), providing another validation of the noise in our SSA implementation.

In total for the well-mixed reaction systems, for 106 of 110 measurements the analytical mean fell within the confidence interval of STEPS output, a success rate of 96% which is approximately equivalent to the 95% success that is expected.

#### ***Diffusion***

Many of the diffusion models could not undergo such precise statistical analysis as the reaction models because in most cases the analytical concentration of an exact position in 1-dimensional axial or radial space is compared to the mean concentration at the center of a small bin of finite tetrahedral volumes in STEPS, which is not a precise comparison. However, simply by visual comparison it could be seen whether STEPS output followed closely what was expected.

**Figure 8:**
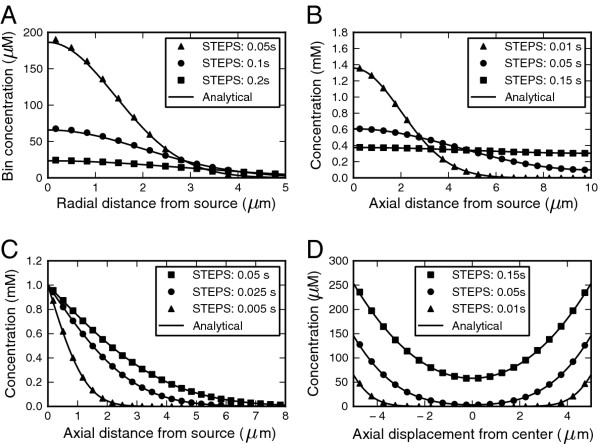
**Validation of diffusion. **In all panels the analytical solution is compared with the mean for 10 (A-C) or 30 (D) iterations of the STEPS simulation at three different times after start of the simulation. **A.** 3D diffusion in an infinite volume from a point source. **B.** 1D diffusion in a finite tube: all the molecules are positioned at the border (distance = 0) initially. **C.** 1D diffusion in a semi-infinite tube with the concentration at the border (distance = 0) clamped. **D.** 1D diffusion in a finite tube with a constant and equal influx of the same species of molecule at both ends (displacement = −5 and +5).

We first tested the most universal case: 3D diffusion from a point source in an infinite volume (Figure 8A), which has a known analytical solution for the time and evolution of the radial mean concentration [47]. While we could ensure the absence of boundary effects, it was not possible to mimic a point source in a tetrahedral mesh. The small deviations between the analytical solution and the STEPS simulation at short distances from the source for early simulation times are due to the finite volume of this source (see Additional file 4). At further distances or later times the match between the mean of the STEPS simulation showed no significant deviation from the analytical solution.

Next we tested three different scenarios for 1D diffusion: from a point source at one end of a finite tube (Figure 8B), in a semi-infinite tube with a clamped concentration at the end (Figure 8C) and in a finite tube with constant influx of the same species at both ends (Figure 8D). These systems each have a known or deduced analytical solution of the time evolution of the axial mean concentration for 1D diffusion, which may in each case be compared to simulation output due to the radial symmetry of the problem. In all three cases the mean of the STEPS simulation and the analytical solution matched closely at all spatial locations for all times.

#### ***Reaction–diffusion***

**Figure 9:**
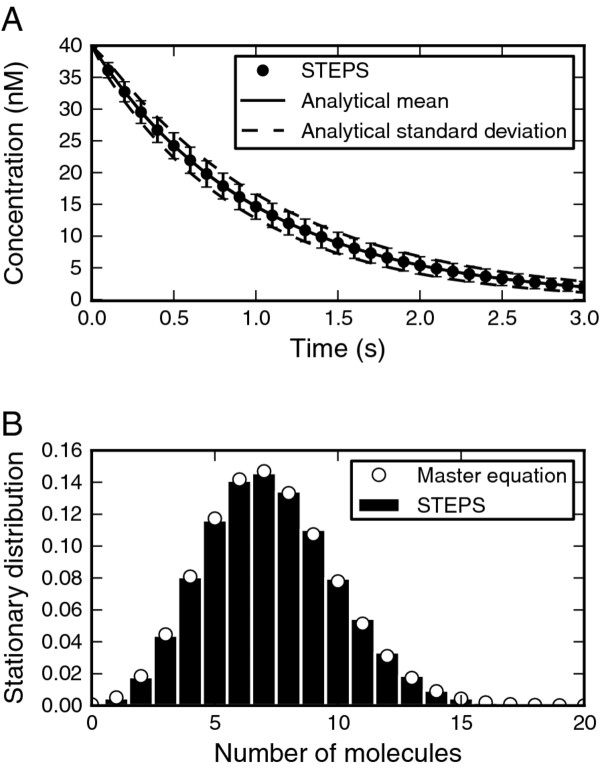
**Validation of reaction–diffusion. A. **Lack of effect of diffusion in STEPS (points with error bars showing the sd) on first-order irreversible reaction (analytical solution, full line) with uniform initial concentration. Setting the diffusion constant to zero did not change the simulation results (not shown). **B.** Diffusion does not significantly affect stationary distribution of molecule that undergoes a zero-order production reaction and second order reaction with clamped species when tetrahedron size is larger than acceptable minimum. STEPS simulation (histogram) compared to analytical solution to chemical master equation (open circles).

In testing the combined simulation of chemical reactions and diffusion we were limited by the paucity of available analytical solutions. A first simple test was to add diffusion to an irreversible first-order reaction with an initial uniform concentration of the reagent. Diffusion should not affect this process, which is confirmed (30 of 30 points fell within 95% confidence interval) (Figure 9A). Next, a very discrete reaction–diffusion problem, typically containing only about 10 molecules in the system, was analyzed. This consisted of two reactions: a zero-order reaction and second order reaction [38]. Ensuring that subvolume size was larger than the accepted lower bound, and significantly larger than the “critical value” discussed in [38], the deviation of the stationary distribution of the reactant from the analytical solution to the master equation was small (Figure 9B).

**Figure 10:**
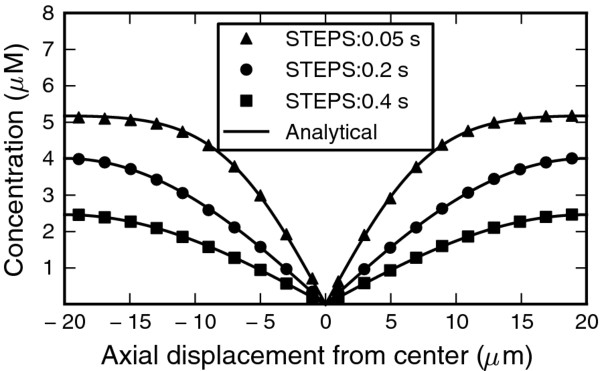
**Validation of a degradation-diffusion reaction. **Two separated reactants diffuse towards each other in the center of a cylinder and annihilate. Comparison of STEPS simulation at different times (symbols) and the analytical solution for clamped diffusion [48].

Finally we present a reaction–diffusion system containing spatial gradients for which we found an analytical solution [48] and where diffusion is important. This is a second-order degradation process where the initially separated reactants diffuse from separate halves of a tube and the assumption that they degrade so fast on contact that their concentration at the center is always zero. The analytical solution then corresponds to diffusion with a concentration clamped at zero, which matched the STEPS simulation closely (Figure 10).

### **Algorithm efficiency**

To make comparisons between the efficiency of the reaction–diffusion algorithm in STEPS to a similar tool we compared to MesoRD [19] and to make comparisons to particle methods we chose Smoldyn since it has been reported to be an efficient particle simulator [12]. All simulations were run on a Macbook Pro 2.4 GHz Intel Core 2 Duo processor and 4 GB 667 MHz DDR2 SDRAM. Care was taken to ensure that the computer performance was as equal as possible for every test, with a measured pystone score of approximately 54500 pystones/second.

Precise comparisons of the subvolume approach employed by STEPS and MesoRD with the particle approach of Smoldyn are difficult due to different factors affecting the efficiency of the two approaches, however we tested a range of simulation conditions with notable comparisons when the simulators are estimated to be at the most efficient with acceptable accuracy. The acceptable spatial resolution in STEPS and MesoRD is estimated as the size at which there are approximately 10 diffusion events per reaction event (of the fastest reaction) per subvolume (see Additional file 2). Smoldyn is not exact for all length time-steps due to the fact that the reactants can only undergo one interaction at the end of a time-step and it is not possible for a molecule to be in existence for less than the length of a time-step. This means that if products are involved in further interactions (as is the case for this model) then errors are introduced for large time-steps. An acceptable time-step in Smoldyn is stated as that at which it is “significantly smaller” than the timescale of the fastest reaction in the system, which we will choose as the time-step that is 10 times lower than the fastest reaction in the system.

In a comparison to the Smoldyn benchmark [12] STEPS appears to perform favorably. This model is a Michaelis-Menten enzyme reaction and the simulation parameters suggest an upper bound of tetrahedron size in STEPS of 2 μm, which corresponds to approximately 140 tetrahedrons in the simulation volume. Simulating in a mesh of 284 tetrahedrons took 10 seconds in STEPS compared to 46 seconds for the Smoldyn simulation (close to the 47 seconds reported on the author’s computer).

The more interesting comparisons in efficiency, however, come in larger models with a greater number of distinct diffusing species with multiple chemical interactions. We ran a test model in STEPS, MesoRD and Smoldyn consisting of 10 molecular species in different concentrations, that diffuse with different diffusion constants, and interact by 8 reaction channels (described in further detail in Additional file 5). STEPS and MesoRD runtime increases with increasing spatial resolution and Smoldyn runtime increases with decreasing time-step, so we compared a range of conditions with an estimate of the point at which the simulators are most efficient yet accurate. All simulations were run 3 times and a median value taken, and in all cases the differences in runtimes between the 3 tests were found to be small.

**Figure 11:**
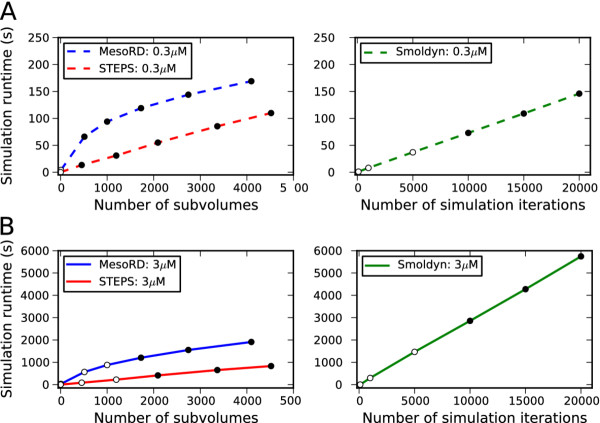
**Simulator efficiency. **Test system simulation runtimes in STEPS, MesoRD and Smoldyn. Filled circles show points where the simulation is calculated to be accurate and open circles show where simulation may be inaccurate. **A.** Low number of molecules initial condition. Left panel: STEPS and MesoRD simulation runtimes at different number of subvolumes describing the same total mesh volume. Right panel: Smoldyn runtimes with varying number of simulation iterations due to a change in time-step. **B.** High number of molecules initial condition, which is the only difference from simulations shown in A. Notice different y-scale between A and B but not between left panels and right panels.

Figure 11 shows simulation times in STEPS, MesoRD and Smoldyn for the test system for two different models, which differ only by the total number of molecules as the initial condition. All simulators are very fast in the most efficient case (very large time-step in Smoldyn, only one volume in STEPS, eight subvolumes in MesoRD) but may be inaccurate. As we then decrease efficiency and increase accuracy we can see that simulation time eventually scales approximately linearly for all simulators, although MesoRD initially scales logarithmically. In the first model a total of 5500 molecules were injected, which corresponds to a concentration of ~0.3 μM, and during simulation the total number of molecules increased to around 6000. The estimated upper limit of acceptable subvolume size in STEPS corresponds to approximately 300 tetrahedrons in the mesh, making the results for a 454 tetrahedron mesh accurate with a simulation time of 13 seconds. The estimate for a cubic mesh puts an acceptable number of cubes at approximately 380 (a small discrepancy from the tetrahedral case arising from the different geometry), giving the fastest simulation with acceptable accuracy at 512 subvolumes in the MesoRD mesh with a simulation time of 66 seconds (it was in fact not possible to generate a 7x7x7 mesh of 343 cubes). The fastest reaction in the system has a characteristic time of 1 ms, so a time-step of 0.1 ms was estimated as the upper-bound for accuracy in Smoldyn with a runtime of 73 seconds. So, at the estimate for the most efficient conditions with acceptable accuracy, STEPS runtime was more than 5 times faster than MesoRD and Smoldyn. As we increase accuracy further in Smoldyn with 10 times more iterations the runtime slows to 726 seconds (not plotted), and with approximately 10 times more subvolumes in STEPS (4520) and MesoRD (4096) STEPS slows to 109 seconds compared to 169 seconds for MesoRD. So, in the lower molecule number case, at the most efficient simulation possible for acceptable conditions STEPS appears to perform favorably, and as we increase detail STEPS appears to maintain an advantage over the other two simulators. At the most detailed meshes tested for STEPS (13871 tetrahedrons) and MesoRD (13824 cubes) runtime was 263 seconds in STEPS and 347 seconds in MesoRD (not plotted).

The spatial SSA approach shows increasing benefit with larger numbers of molecules. In the second model a total of 55000 molecules were injected, which corresponds to a concentration of ~3 μM, and during simulation increased to around 60000. The upper-limit of acceptable spatial resolution was estimated to correspond to approximately 1200 tetrahedrons in the mesh for STEPS and 1500 cubes in the MesoRD mesh, and the fastest reaction time in Smoldyn at the start of the simulation remained 1 ms. The STEPS simulation with 2090 tetrahedrons took 413 seconds compared to 1205 seconds in a mesh of 1728 cubes in MesoRD. In Smoldyn, with a time-step of 0.1 ms, simulation time was 2857 seconds. With approximately double the number of subvolumes in STEPS (4520) and MesoRD (4096) runtimes increased to 831 seconds and 1910 seconds respectively. With twice the number of iterations in Smoldyn (time step of 0.05 ms) simulation time was 5745 seconds.

Direct comparison between STEPS and MesoRD show that STEPS performs significantly better in all tested conditions. Direct comparisons to Smoldyn are not possible, however something that can clearly be seen is that the magnitude of the slopes between the two different initial conditions means that, for a factor of 10 increase in the number of molecules, STEPS slows by a factor of approximately 8, whereas Smoldyn slows by a factor of approximately 33 and this factor is expected to become even larger in models with a greater numbers of molecules. Notice, however, that a higher molecule number does mean a slightly finer mesh must be used in STEPS and MesoRD to ensure the well-mixed subvolume condition.

### **Importing SBML models**

STEPS provides thorough and well-validated support for SBML [21], a common format for representing biochemical models. STEPS has been tested to successfully run the majority of the SBML Test Suite models and “curated” models from the BioModels Database [49], with results validated against published solutions. The high-level of support has largely been made possible by supporting MathML expressions. Such expressions, which are very common in SBML models, often include simulation variables that must be stored and available to use by the solver. STEPS stores the MathML expressions in Python structures, updates variables in the expressions during simulation, and can solve the expressions whenever necessary. This has been crucial for STEPS support for SBML components such as Function Definitions, Initial Assignments, Assignment Rules, Rate Rules, Event Triggers and Event Assignments. For the special case of Reactions, STEPS is able to examine the form of the Reaction Kinetic Law maths and separate them into two categories: those that can be represented as an ordinary reaction in STEPS, and those that must be solved by an approximate method because the Kinetic Law maths differs from a fundamental reaction (many models contain at least one of these types of reaction). This ensures that a reaction will never be represented incorrectly in STEPS. Any simulator, such as MesoRD, that does not provide thorough MathML support is limited in its support of many SBML components, and any stochastic simulator that does not examine the form of Reaction Kinetic Law maths will represent most published models incorrectly. STEPS support extends to models containing multiple compartments and surfaces, along with volume-surface and surface-surface reactions. In the end STEPS successfully imported 654/980 SBML Test Suite 2.0.0 l3v1 models (Additional file 6: Figure S1). Solutions are provided with the Test Suite for every model, so by automated testing a large number of models could be imported, simulated in the deterministic Wmrk4 solver in STEPS and results compared against given solutions. Of the 326 models that failed 262 fell into 3 categories, those that contained: (1) 0-dimension or 1-dimension compartments that are not supported in STEPS, (2) no chemical species (STEPS requires at least one chemical species in order to run a simulation) and (3) Algebraic Rules, which are difficult to support in a STEPS context and rarely appear in SBML models. The other models that failed included some that are not possible to run in a discrete stochastic context such as those that include partial stoichiometry or negative concentrations and as such are also not supported in the STEPS deterministic solver, which shares model construction with the stochastic solvers. STEPS also successfully imported 223/326 curated BioModels Database models (downloaded in June 2011), where possible the most recent versions of each model (often l2v4) were imported. The majority of the failures were again because of no chemical species in the model, partial or high reactant stoichiometry, and also included those with unsupported units such as amperes or volts.

**Figure 12:**
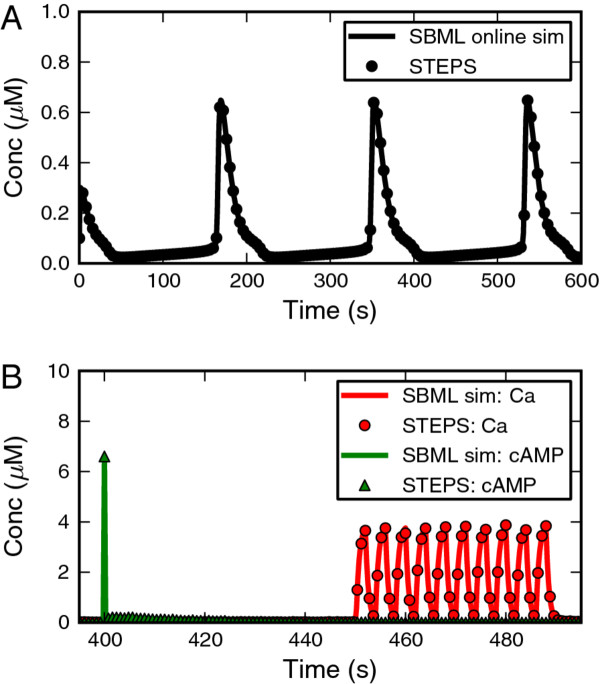
**Example simulations. A. **SBML model of calcium oscillations in astrocytes simulated in STEPS with the deterministic solver Wmrk4 (results plotted at 4 second intervals) shows perfect agreement with BioModels Online simulation. **B.** SBML model in a femtoliter compartment is simulated in STEPS with the stochastic solver Wmdirect (results plotted at 0.8 second intervals) and compared to BioModels Online deterministic simulation. Shown are cAMP and Ca signals, which are controlled by Events. Small stochastic differences are noticeable between simulations.

Imported models may be directly simulated in STEPS using the deterministic solver or the well-mixed stochastic solver, although many models are not suitable for stochastic simulation without some modification. It is worth noting that SBML models may potentially form the basis of simulations in the spatial stochastic solver, but not without some modifications; for example diffusion coefficients and non-uniform initial conditions must currently be defined outside of SBML.Figure 12A shows a deterministic simulation in STEPS of model BIOMD0000000184 from the BioModels Database in comparison to a BioModels Online Simulation. This model of spontaneous calcium oscillations in astrocytes [50] contains two compartments (cytoplasm and endoplasmic reticulum) with transport reactions between them. Some reactions in the model can be represented as fundamental reactions, but some reactions contain complex maths, which is therefore converted to Python structures allowing for solution by the approximate method. Figure 12A shows close agreement between the STEPS simulation and the BioModels Online Simulation, and also matches the published results [50] (not shown).

Some SBML models are suitable for well-mixed stochastic simulation without any modification. One example is BIOMD0000000152, which contains a femtoliter compartment and micromolar concentrations. As described further in [51] this is a larger model containing 63 Species and 120 Reactions, and also 21 Events which represent ‘cAMP’ and ‘Ca’ signals. At time 400 s a pulse of cAMP is introduced, and then intermittently between times 450 s and 490 s the reaction parameter controlling calcium influx is toggled between a low and high value by Events. When running the simulation in STEPS the mathematics representing Event Triggers and Event Assignments is stored in Python structures, which makes it possible to represent these important features of the model. Figure 12B shows simulation results using the STEPS stochastic solver superimposed on deterministic results from a BioModels Online Simulation, displaying the two species directly involved in Events: cAMP and Ca. STEPS matches the BioModels Online Simulation results, with some small expected variability arising from the stochastic simulation.

## **Discussion**

### **Advantages and disadvantages**

As described by example in [22] the Python interface to STEPS brings a number of advantages over reaction–diffusion simulators that have a non-interactive interface. STEPS modelers have greater freedom and control over a simulation, and may utilize the many powerful scientific tools already available for Python for tasks such as data analyses and visualization. In fact we believe a powerful interface is the *only* way to be able to achieve the complex tasks that go with initializing, running and collecting data from simulations in complex 3D geometries.

STEPS provides substantial functionality (significantly more than is often available in similar software) in the Python interface that a modeler may use for building a model and customizing a simulation, which can be particularly important for spatial simulations. Examples are the steps.geom.Tetmesh class functions that are crucial for acquiring and utilizing information about complex 3D mesh geometry, and the functionality in the STEPS Tetexact solver that allows for the manipulation of molecule counts, reaction and diffusion rates, both compartment and patch-wide or individually for tetrahedrons and triangles. There are many possible applications for such functionality, including complex initial conditions [22], chemical localization, control over reaction rates by external variables such as voltage or temperature, and support for some of the more advanced features of SBML such as Rules and Events. All functions are described in detail in the user documentation.

Python is becoming particularly important in the neurosciences and as more and more neural simulators adopt a Python interface the future may even see Python used to glue simulators together so that phenomena on different spatial scales can be integrated, although there may be more efficient alternatives [52]. The Python interface is an advantage now and as biological simulation becomes more and more complex, will be an advantage for the future.

STEPS is capable of accurately representing complex boundaries by supporting unstructured tetrahedral meshes. We demonstrated that while the regular cubic meshes supported by some other simulators are very easy to generate they limit the morphological resolution. Tetrahedrons are able to adapt their shape and size to regions of high morphological detail, which means that such meshes (with sufficient spatial resolution) are able to follow the complex boundaries of a cell very closely. Tetrahedrons may be larger for regions of less interest, which is an important consideration for simulator efficiency. However, with tetrahedron-based geometry comes the difficulty of generating high-quality meshes, which is a drawback for this approach. Specialist mesh generation software is best left to this task and there are some powerful tools available, with common output formats supported by STEPS. Complex boundary generation is not a unique problem to STEPS and particle-based simulators that support complex surfaces, which may for example be represented by collections of triangles or squares, may also require outside software to develop sufficient quality surfaces.

STEPS has the advantage of supporting both spatial and non-spatial stochastic simulations as well as deterministic simulations, and in the near future will even be able to combine spatial and non-spatial compartments in the same stochastic simulation. This may be a very important feature for efficiency in some models while still allowing for complex boundary representation. For example, if one wished to simulate calcium release from intracellular calcium stores in the endoplasmic reticulum (ER), it would be vital to represent the resulting calcium gradients in the cytoplasm in a spatial compartment, yet the simulation would be severely slowed by simulating diffusion in the highly-concentrated ER, which may only have a negligible effect on outcome. Representing the ER as a well-mixed compartment would reduce runtime considerably without affecting accuracy.

### **Chemical accuracy of reaction–diffusion algorithms**

Any representation of a biochemical system on a computer is of course not an exact replication of the real system. Simplifications are made, both by the modeling software and in the designed model itself, to ensure that the problem is tractable and may be simulated on the limited computational power available in an acceptable amount of time. For example, many approaches to biochemical reaction diffusion simulation ignore the crowded environment of the cell formed by macromolecular structures, a feature that can have a significant affect on apparent diffusion coefficients and reaction rates [44,53]. Every approach to incorporating spatial detail into stochastic biochemical simulations makes a different set of simplifying assumptions, which means that the most accurate approach may depend on the properties of the simulated model.

A popular approach to reaction–diffusion modeling is based on Smoluchowski theory [54], which tracks diffusing point-like particles that may react when they fall within a certain distance of another reactive molecule. This theory has the advantage of including some consideration of molecule size, but is limited by its own simplifying assumptions about the system, which can lead to small errors if, for example, two reactants are at a similar concentration to each other [55,56], which is of course often the case for biological systems. In addition, the theory is derived for a system with only one reaction present, making results for all systems with more than one reaction approximate. However, a small time-step can often ensure good accuracy, but this comes at a cost to efficiency.

The spatial Gillespie approach makes the assumption that the subvolumes represent well-mixed regions and, as we have seen, at relatively very large or very small sizes this may not be the case, so some care must be taken by the modeler to ensure subvolumes are in the well-mixed range. A benefit of this approach is the potential gain in efficiency, but a drawback is that it is as yet unclear how to incorporate molecule size and macromolecular crowding into simulations, although this may be possible in the future. The unstructured mesh in STEPS already allows for complex boundaries that could potentially form impenetrable fixed structures within the volume that could go some way towards replicating the crowded environment of the cell. The incorporation of reactant size into the Gillespie framework is an active area of research and it has already been found that for a unimolecular second-order reaction system in one dimension reactant size may be simply incorporated by replacing system volume with the “free volume” [57] and in two dimensions the propensity function is still applicable, yet with a larger correction than just the free volume [58]. This suggests an extension to 3 dimensions will involve a correction to the propensity function based on the excluded volume from the molecules, which could potentially be found from a fixed user-defined parameter of molecule size. Allowing the molecule size to be defined explicitly could ensure that it is always biologically feasible. Where the implied molecules size is found intrinsically from reaction and diffusion parameters (as is the case for Smoluchowski models) the calculated binding radius can be very different from the physical size of the molecules; typical reaction and diffusion parameters of proteins give a binding radius that is unrealistically small [38], and slow diffusion with a fast reaction can lead to a binding radius that is very large. In addition to this possibility, one intriguing approach to this problem is to apply a hybrid method where discrete and continuous spatial descriptions are both permitted, and the simulator combines the spatial Gillespie method and Brownian dynamics [59].

Despite their limitations, current voxel-based and particle methods can often both be shown to be good approximations for a range of biological conditions and a lot of useful information about many systems can be extracted from their simulation. In the future, as computational power increases, our understanding of cellular systems improves and new algorithms are developed (or existing algorithms modified), biochemical computing will surely become more and more powerful and accurate. Only time will tell how significant these future improvements will be, or whether current methods are accurate enough for most studies. At present, with different methods for stochastic reaction–diffusion simulation within complex boundaries essentially producing the same results for a wide range of biological conditions, software efficiency, reliability and ease of use are often the most important considerations for a modeler.

### **Software efficiency**

The core algorithm in STEPS is an efficient implementation of the spatial Gillespie approach to reaction–diffusion modeling and contains the potential for further improvements to runtime in the future with the introduction of approximate methods such as tau-leaping [60] adapted for diffusion [61,62]. Efficiency in a spatial Gillespie simulation depends on the number of mesh subvolumes so care must be taken to ensure that, where possible, the subvolumes are close to the upper bound of accepted size, as discussed in Subvolume size.

STEPS performed favorably in direct comparison to another subvolume-based simulator, MesoRD, in a wide range of conditions, which demonstrates the efficiency of the STEPS implementation. Although it is difficult to precisely compare simulator efficiency between spatial Gillespie and particle methods, partly because of the difficulty of pinpointing the exact point at which the simulator becomes accurate, what can clearly be seen by comparison to the efficient particle simulator Smoldyn is that the spatial Gillespie method in STEPS has a significant advantage over particle methods at higher molecule numbers. Also, once accurate conditions have been met, STEPS performs better than Smoldyn with increasing spatial resolution in STEPS compared to decreasing time-step and increasing accuracy in Smoldyn. This is important because simulation conditions are often more detailed than the very upper-bound of acceptable conditions so as to ensure accuracy or because of complex boundary restrictions.

An important point is that voxel-based software such as STEPS only begins to lose accuracy when subvolumes become very small (as discussed in Subvolume size) and efficiency is low, whereas, conversely, accuracy in particle methods generally increases with smaller time-step and therefore lower efficiency. This means that spatial SSA methods are most accurate at efficient simulation conditions, whereas particle methods are generally most accurate at inefficient conditions.

### **Validation**

Regardless of the approach and the capabilities of the simulator it is important that all supported features are validated by analyzing output, where possible comparing to known analytical solutions. Validation ensures that there are no numerical errors resulting from bugs in the code so that the software may be reliably used for research purposes, and repeating this validation every so often checks that any recent changes to code have not resulted in loss of accuracy. STEPS is well tested and validated for the majority of its capabilities as we have reported in this paper, yet other simulators are often poorly validated and may be unreliable, particularly when it comes to capturing the noise resulting from stochastic chemical reactions. As reaction–diffusion simulators become more and more widely used to investigate the molecular properties of neural and other biochemical systems it is vital that each simulator is known to be reliable and accurate. For this reason it is important that standards for validation for reaction–diffusion simulators are developed, as has been achieved for example for the electrical properties modeled by neuronal simulators [63]. The set of validations that we have presented in this paper could contribute towards such a future reaction–diffusion standard.

### **The future**

The future will see further additions to STEPS as more and more biological phenomena are added to models. In neurons in particular, the intracellular signaling pathways are highly coupled to the electric excitability of the cell through the activity of voltage-gated channels on the membrane. A powerful addition to future versions of STEPS will be the calculation of the potential across surfaces representing membranes within the tetrahedral mesh geometry, to which voltage-gated ion channels may be added. In the near future lateral diffusion will also be implemented to simulate the mobility of molecules in membranes. Further ahead, one possibility is to allow meshes to dynamically alter their shape during simulation to replicate real changing cell shape. One potential application for this is the simulation of the enlargement of dendritic spines associated with long-term potentiation [64,65].

Future additions to STEPS will also be based on considerations of efficiency. Since spatial simulations are mainly dominated by diffusion, the largest gain in efficiency may come with implementing approximate methods for diffusion.

Currently STEPS is developed and tested under 32-bit systems, thus the simulation size is restricted by 4 GB of addressable memory, approximately 10^8^ kinetic processes. Although this restriction can be eased by converting STEPS to a 64-bit version, the simulation of very large scale systems on individual workstations can be impractical due to a long runtime. Solution of this problem will depend on the development of an efficient parallel framework, where the whole system is distributed and simulated in different nodes of a computing cluster. A great challenge for such a parallel framework is the need to reduce network communication as well as preventing unnecessary rollbacks caused by state conflicts between nodes.

## **Conclusions**

Discreteness, stochasticity and spatial effects are vital considerations for capturing the dynamics of many cellular molecular systems, yet this high level of detail makes efficiency a particularly important consideration for tools such as STEPS that are designed to simulate such systems. Efficiency is tied to accuracy, gains in one often coming at a cost to the other. STEPS employs the spatial SSA approach to discrete reaction–diffusion simulation, which is generally more efficient than particle-based methods, yet more abstracted conceptually. However, we have shown that there is usually no loss or a minimal loss of accuracy for biochemical systems, provided that due consideration is given to subvolume size. The optimized algorithm in STEPS was shown to out-perform both another SSA-based simulator, MesoRD, and particle methods by comparison to Smoldyn, with increasing benefit in larger systems and increasing simulation detail. In terms of spatial accuracy, STEPS offers improvement over other spatial SSA software by supporting tetrahedral meshes, which provide higher morphological resolution than cubic voxels. The problem of representing complex boundaries in surfaces or meshes is best left to powerful, specialist software, and common formats are imported by STEPS. The distinction between biochemical model, geometry description and solver method offer a number of advantages, such as the ability to apply different simulation techniques to the same model, and to reuse complex geometry descriptions. Solver accuracy was confirmed in an extensive validation suite consisting of a set of reaction, diffusion and reaction–diffusion systems. The Python interface to STEPS was found to play an important role in almost all aspects of creating models, running test simulations and building additional features, including reliable support for SBML.

Therefore, STEPS successfully combines both high performance and high accuracy within a powerful and user-friendly interface, allowing application to a large number of biochemical network models where stochasticity and spatial organization play a prominent role. The framework in STEPS offers the potential for future improvements to performance, such as approximate method implementation and parallelization, which will add more power and open up even more applications as larger systems may be simulated. Further additions to the code will open up the exciting possibility of full integration with the electrical properties of the cell, allowing accurate and efficient parallel multi-scale neural simulations. For these and further future additions, which could for example potentially include new algorithms to represent the crowded environment of the cell, scientific accuracy and software efficiency will both continue to play prominent roles in STEPS development.

## **Availability and requirements**

**Project name:** STochastic Engine for Pathway Simulation

**Project home page:**http://steps.sourceforge.net

**Operating system(s):** Platform independent

**Programming languages:** C/C++, Python

**Other requirements:** Python 2.5 ~ 2.7

**License:** GNU General Public License version 3

Source code and pre-compiled binaries for Windows and Mac OS X are available at the home page where further information about STEPS can also be found, including the Online User Manual.

## Competing interests

The authors declare that they have no competing interests.

## **Authors’ contributions**

IH participated in STEPS development, was the main author of the SBML import module, carried out validation, geometry accuracy and software efficiency studies, and drafted the manuscript. WC was involved in STEPS development, including implementation of the composition and rejection method, and performed all mesh constructions. SW conceived the STEPS workflow and carried out all the original STEPS development. EDS conceived of and supervised the project and helped draft the manuscript. All authors contributed to the manuscript and read and approved the final version.

## Supplementary Material

Additional file 1 **Gillespie Stochastic Simulation Algorithm (SSA), optimizations and implementations in STEPS.** A description of the algorithms and optimizations used in STEPS solvers Wmdirect and Tetexact [66,67]. Click here for file

Additional file 2 **Subvolume Size. **An analysis of acceptable tetrahedron size range for reaction-diffusion simulations in STEPS. Click here for file

Additional file 3 **Cubic and Tetrahedral mesh comparison. **A statistical comparison between cubic and tetrahedral mesh representation of five different dendritic spine geometries. Click here for file

Additional file 4 **Validation. **Detailed descriptions of the validation models used in this study, along with statistical analysis of results from STEPS simulations. [68]. Click here for file

Additional file 5 **Simulator Efficiency Test Model. **A description of the model used to study efficiency of STEPS, MesoRD and Smoldyn, along with model files for each simulator. Click here for file

Additional file 6 **Figure S1. SBML Test Suite support. **All 980 models (l3v1) of the SBML Test Suite 2.0.0 (as of 2011/06/01) were imported, run in the Wmrk4 deterministic solver in STEPS and results compared against given solutions. The chart shows the proportion of models supported (red) and unsupported (other colors). The unsupported models are separated into 4 categories. Click here for file
