# Supplementary material for: STEPS: efficient simulation of stochastic reaction–diffusion models in realistic morphologies
Source: BMC Syst Biol. 2012 May 10;6:36. doi: 10.1186/1752-0509-6-36 (PMC3472240; doi:10.1186/1752-0509-6-36)
Supplement: Additional file 1 — Gillespie Stochastic Simulation Algorithm (SSA), optimizations and implementations in STEPS. A description of the algorithms and optimizations used in STEPS solvers Wmdirect and Tetexact [66,67]. [file 1752-0509-6-36-S1.pdf]

1. Gillespie Stochastic Simulation Algorithm (SSA), optimizations and implementations in STEPS

In a system where molecules are uniformly distributed and their velocities are thermally randomized to the Maxwell-Boltzman distribution the position and velocity of individual molecules can be ignored and one can describe the probability of the system moving from one discrete state **x**, a vector listing the number of molecules present for *N* species, to another state accurately. These chemical transitions are governed by the propensity function a_j, defined so that $a_j(\mathbf{x})dt$ is the probability that one reaction R_j out of *M* possible reactions will occur in the next infinitesimal time interval for a specific state $\mathbf{x}_t$. The propensity function was first derived for dilute gases [17] and, importantly for cellular systems, has recently been derived for solute molecules in a bath of smaller solvent molecules [66]. The zero propensity a_0 is the sum of all propensities and gives the probability that any reaction will occur in the infinitesimal time interval. The SSA uses these propensity functions to compute when the next reaction occurs. The algorithm applies to irreversible reactions but a reversible reaction can always be described as the combination of two irreversible ones.

Different variations of the SSA exist, mostly originate from the Direct Method, which can be summarized as:

1. Set $t = 0$ and initialize the state $\mathbf{x} = \mathbf{x}_0$.
2. Evaluate $a_j(\mathbf{x})$ for all reactions $1 <= j <= M$ and compute $a_0(\mathbf{x})$.
3. Draw a random number r_1 from the unit-interval uniform distribution.
4. Generate τ, which is the time interval until the next reaction event using the equation $\tau = \frac{1}{a_0(\mathbf{x_t})}\ln\left(\frac{1}{r_1}\right)$. If $t + \tau > t_{end}$ set $t = t_{end}$ and quit the loop.
5. Draw another random number r_2 from the unit-interval uniform distribution. Find the index *j* of the next reaction R_j by performing a linear search along the propensity values until the condition $\sum_{j=1}^{j-1} a_j(\mathbf{x_t}) < r_2 * a_0(\mathbf{x_t}) \le \sum_{j=1}^{j} a_j(\mathbf{x_t})$ is met.
6. Apply reaction R_j to $\mathbf{x}_t$. Update all affected propensities and recompute $a_0(\mathbf{x})$.
7. Set $t = t + \tau$.
8. Go back to step 2 for another iteration.

1.1 Well-mixed solver

Optimized data structure and searching/updating algorithms are essential to the performance of SSA simulators. The original Direct method [17] performs linear searching and updating on an array of propensities, giving an overall computational complexity of O(*M*). It was later enhanced by Gibson and Bruck ([28], section 7.2) using a binary tree structure and a dependency graph of kinetic processes. In the enhanced version, each leaf of the binary tree stores a propensity value. The values of each pair

of sibling nodes, i.e. nodes sharing the same parent node, are summed and stored in their parent node iteratively

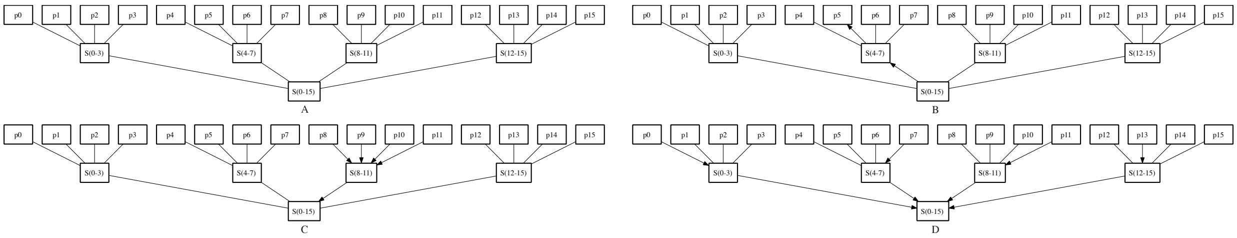

until a single "root" node is produced, which stores a_0 (the zero-propensity). The entire tree contains $\log_2 M$ levels, thus can be stored in $2M$ memory space. With the binary tree structure, both searching and updating can be performed in a logarithmic time scale as $O(\log_2 M)$. This enhancement is adapted in the implementation of STEPS. Furthermore, to make use of modern hardware, we extend the binary tree to k-ary tree, which provides $O(\log_k M)$ complexity as well as better caching performance (above figure, A). The search of the next kinetic process is performed bottom-up, i.e., from the root node to its descendant leaves (B), while the updating is performed top-down (C, D). A significantly high percentage of cpu time is spent on the updating of propensities [67] and it is important to optimize this process. To update the propensities of E kinetic entries, the Gibson and Bruck method requires updating partial sums of $\log_k M$ - 1 branches of the k-ary tree in the best case, when all changing entries have the same parent node (C), compared to $E\log_k M$ - 1 branches in the worst case, if neither the updating entries nor their ancestor nodes are siblings until the root node is reached (D).

We replace steps 5 and 6 in the Direct Method with:

5. **[STEPS 1.3: Wmdirect]** Draw another random number r_2 from the unit-interval uniform distribution. Search the k-ary tree to find the index j of the next reaction R_j that meets the condition $\sum_{j=1}^{j-1} a_j(\mathbf{x_t}) < r_2 * a_0(\mathbf{x_t}) \le \sum_{j=1}^{j} a_j(\mathbf{x_t})$.
6. **[STEPS 1.3: Wmdirect]** Apply reaction R_j to $\mathbf{x}_t$. Update affected branches of the k-ary tree.

1.2 Spatial solver

In version 1.3 the implementation of Tetexact solver adopts the Composition and Rejection (CR) Method [31], whose time complexity is constant even for systems with large number of reactions. In the CR Method, reactions are grouped by their propensity magnitudes. For each SSA iteration, the CR Method firstly select a group g by linear or binary search so that $\sum_{g=1}^{g-1} sum_g(\mathbf{x_t}) < r_2 * a_0(\mathbf{x_t}) \le \sum_{g=1}^{g} sum_g(\mathbf{x_t})$, where sum_g is partial sum of propensities in g. In most of the simulations the total number of groups, G, is bounded and independent of the number of reactions in the system, thus the time of above

searching can be considered as constant. After a group is picked, a constant time rejection sampling is performed to select the next suitable reaction from this group. Updating time is also constant by a carefully designed data structure and algorithm. The search and update of the CR Method can be described as:

5. **[STEPS 1.3: Tetexact]**
   5.1. **Composition:** Draw another random number r_2 from the unit-interval uniform distribution. Find the group *g* that meets the condition
   $\sum_{g=1}^{g-1} sum_g(\mathbf{x_t}) < r_2 * a_0(\mathbf{x_t}) \le \sum_{g=1}^{g} sum_g(\mathbf{x_t})$.
   5.2. **Rejection:** In group *g* with *k* reactions, draw a uniform random integer *i* from 1 to *k*, and a uniform random number a_r from a^g_{min} to a^g_{max} which are the minimum and maximum of propensities in group *g.* If $a_r < a_i^g(\mathbf{x_t})$, go to step 6, else repeat step 5.2.
6. **[STEPS 1.3: Tetexact]** Apply reaction R^g_i to $\mathbf{x}_t$. Update affected groups and recompute $a_0(\mathbf{x})$ from group sums.
