# Supplementary material for: STEPS: efficient simulation of stochastic reaction–diffusion models in realistic morphologies
Source: BMC Syst Biol. 2012 May 10;6:36. doi: 10.1186/1752-0509-6-36 (PMC3472240; doi:10.1186/1752-0509-6-36)
Supplement: Additional file 2 — Subvolume Size. An analysis of acceptable tetrahedron size range for reaction-diffusion simulations in STEPS. [file 1752-0509-6-36-S2.pdf]

2. Subvolume Size

Upper bound
We can state that the subvolume is well-mixed if the time-scale of diffusion for reactants is significantly smaller than the time-scale of the fastest reaction within the volume. This ensures that spatial gradients resulting from a reaction event are removed by diffusion long before the next reaction.

For example, consider that the fastest reaction in the system is a second-order reaction system:

$$A + B \xrightarrow{k} C \tag{1}$$

The typical timescale of the reaction is given by:

$$\tau_k \simeq \left(kN_A V[A][B]\right)^{-1} \tag{2}$$

where k is the reaction constant (in units of liter/mol.s or similar), N_A is Avogadro's number, V is the volume of the subvolume, and $[A]$ and $[B]$ are the concentrations of the reactants. Strictly speaking, this timescale applies at relatively large sizes when there are enough molecules per tetrahedron that we may talk about the molecule concentrations and ignore discreteness.

The timescale for diffusion of a molecule within the volume is approximately:

$$\tau_D \simeq \frac{h^2}{D} \tag{3}$$

where h is the "size" of the volume (in dimensions of length) and D is the diffusion constant of the molecule.

However, if we define h as the edge length of a tetrahedron (which is the simplest mathematically) we may overestimate the diffusion time by an order of magnitude or so with the above assumption because the edge-length is the largest size possible by which to define a tetrahedron. Approximating the diffusion time as the time for a molecule to diffuse through one face of a regular tetrahedron to a neighboring tetrahedron it turns out:

$$\tau_D \simeq \frac{h^2}{9D} \tag{4}$$

For our well-mixed assumption to hold we require:

$$\tau_D < \tau_k \tag{5}$$

which gives:

$$h < \left(\frac{108}{\sqrt{2}} \frac{D}{kN_A[A][B]}\right)^{1/5} \tag{6}$$

(assuming h is the edge length of a regular tetrahedron, then $V = \frac{\sqrt{2}}{12}h^3$).

We should bear in mind that our base volume units are now the cubic meter (not to be confused with the liter).

Note that the subvolume size does not have to be “much smaller than” this size, but should be such that the number of diffusion events should be “significantly larger” than the number of reaction events locally. For example, the point at which we would have roughly 10 diffusion events per reaction event would be:

$$10\tau_D = \tau_k \tag{7}$$

which gives:

$$h = \left(\frac{108}{10\sqrt{2}} \frac{D}{kN_A[A][B]} \right)^{1/5} \tag{8}$$

so the $10^{-1/5}$ factor means that we only have to go to about 60% of the original size estimate to insure approximately 10 diffusion events per reaction.

In real reaction systems our fastest reaction may be a first order reaction, and our size consideration would be:

$$h < \left(\frac{108}{\sqrt{2}} \frac{D}{kN_A[A]} \right)^{1/5} \tag{9}$$

or ensuring 10 diffusion events per reaction event:

$$h < \left(\frac{108}{10\sqrt{2}} \frac{D}{kN_A[A]} \right)^{1/5} \tag{10}$$

where [A] is the concentration of the reactant.

Equations 8 and 10 are used to calculate the upper-bound estimate for simulations in this paper. We should note at this point, that with a uniform initial distribution if our tetrahedrons were larger in volume than the upper bound we would of course not see any deviation in behavior from the well-mixed case and we might be tempted to say, therefore, that there is no upper bound on tetrahedron size. However, this is only in the case of a uniform initial distribution, which is not, generally speaking, what 3D spatial stochastic simulations are designed for. The reason that we impose this upper bound is to correctly represent spatial gradients in the system with good resolution in realistic simulations, which would otherwise be lost in coarser meshes. The estimate for upper-bound should strictly speaking be taken for the conditions during the simulation that would give the smallest size, i.e. at the point at which concentrations are at their highest.

Lower bound

At a certain point the mean concentration per subvolume becomes less than one molecule meaning that the number of molecules inside a particular tetrahedron in the simulated system is usually one or zero, and this discreteness affects the time-scales

for reactions inside subvolumes compared to the continuous description. Considering the discrete case results in an estimate of the minimum subvolume size mathematically, but one should also bear in mind more physical considerations such as molecule size and the mean-free path.

If we are considering the same second-order reaction between chemical species A and B, but this time in a tetrahedron containing only one molecule of reactant A and one molecule of reactant B then the time-scale of the reaction is now:

$$\tau_k \approx \frac{N_A V}{k} \tag{11}$$

So in the discrete case the reaction time is proportional to volume (which is of course proportional to the cube of the subvolume size), which means that as volume decreases the reaction time decreases faster than the diffusion time, which is proportional to the square of the subvolume size. Even though we only have one species of each reactant molecule in the subvolume, by a number of arguments we must still maintain the condition that diffusion time is significantly smaller than reaction time. The well-mixed assumption remains that there are many more elastic (non-reactive) collisions inside subvolumes than reactive ones and if reaction time is smaller than diffusion time we are clearly violating that condition for the reactants and perhaps also for any products from the reaction. Moreover, if we get to the point at which two reactants are more likely to react inside than diffuse from any given subvolume then the simulated system algorithmically becomes dominated by diffusion. If we were to decrease size further and further then diffusion more strongly dominates the system meaning that results would differ with different mesh sizes. Also, if a reaction is more likely than diffusion from the subvolume this implies we may be approaching the size of the molecules themselves, violating our condition that molecules don't occupy a significant proportion of the volume. In short, our condition remains that we wish the diffusion time from a tetrahedron to be smaller than reaction time when one molecule of each reactant is present inside the subvolume, which gives a minimum tetrahedron size.

So our condition remains (equation 5):

$$\tau_D < \tau_k$$

which is, in the discrete case (equations 4, 11, 5 and $V = \frac{\sqrt{2}}{12} h^3$ as before):

$$\frac{\sqrt{2}}{12} \frac{N_A h^3}{k} > \frac{h^2}{9D} \tag{12}$$

rearranging:

$$h > \frac{12}{9\sqrt{2}} \frac{k}{N_A D} \tag{13}$$

or approximately:

$$h > \frac{k}{N_A D} \quad (14)$$

which we may consider as our first estimate for lower limit for tetrahedron size, if h is the side-length of a regular tetrahedron as before. This is approximately equivalent to the "critical value" found in [38].

A better estimate would be the size at which there are approximately 10 diffusion events for every reaction event locally, to ensure reaction time and diffusion time are not just comparable, but that diffusion dominates over the reaction. This also ensures the size is significantly larger than the "critical value" and, therefore, no correction to reaction rates are necessary:

$$h > \frac{120}{9\sqrt{2}} \frac{k}{N_A D} \quad (15)$$

or approximately:

$$h > \frac{10k}{N_A D} \quad (16)$$

We take equation 16 as our estimate for the lower bound of tetrahedron size for this paper.
