# Supplementary material for: STEPS: efficient simulation of stochastic reaction–diffusion models in realistic morphologies
Source: BMC Syst Biol. 2012 May 10;6:36. doi: 10.1186/1752-0509-6-36 (PMC3472240; doi:10.1186/1752-0509-6-36)
Supplement: Additional file 3 — Cubic and Tetrahedral mesh comparison. A statistical comparison between cubic and tetrahedral mesh representation of five different dendritic spine geometries. [file 1752-0509-6-36-S3.pdf]

# 3. Cubic and Tetrahedral mesh comparison

We generated 5 geometries which consisted of a sphere attached to a cylinder to approximate dendritic spines. Sizes were constrained by data from [46] and chosen to represent a good range of between the given bounds of head (sphere) radius, neck (cylinder) length and neck radius (see figure below). For each geometry two cubic meshes were generated in MesoRD [19], and two adaptive tetrahedral meshes were generated in CUBIT [32], with the number of subvolumes in the cubic and tetrahedral meshes similar to each other in both the larger and smaller subvolume cases. The mesh statistics are given below, compared to the ideal values. All sizes are based on microns:

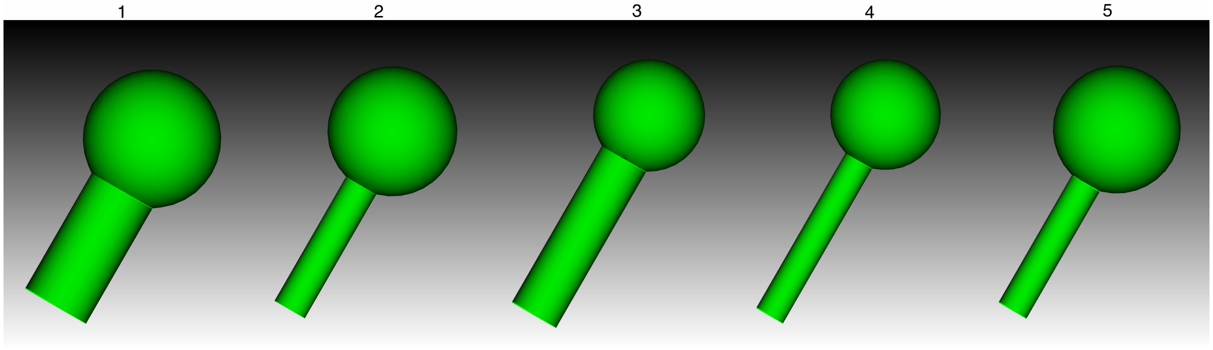


| **Spine #1:** | Head radius: 0.285153 | Neck radius: 0.144017 | Neck length: 0.544474 | | | |
|---|---|---|---|---|---|---|
| Mesh | Subvolume size | Subvolume # | Head volume | Neck volume | Head surface area | Neck surface area |
| Ideal | n/a | n/a | 0.0958199 | 0.0354774 | 0.951852 | 0.492685 |
| Cubic 1 | 0.035 | 3080 | 0.096383 | 0.0356720 | 1.43570 | 0.627200 |
| Tetrahedral 1 | adaptive | 3052 | 0.0963011 | 0.0357806 | 0.957998 | 0.500006 |
| Cubic 2 | 0.022 | 12432 | 0.0965987 | 0.0357773 | 1.44619 | 0.650496 |
| Tetrahedral 2 | adaptive | 12527 | 0.0959651 | 0.0354396 | 0.953679 | 0.494545 |
| | | | | | | |

| **Spine #2:** | Head radius: 0.302343 | Neck radius: 0.0808531 | Neck length: 0.663360 | | | |
|---|---|---|---|---|---|---|
| Mesh | Subvolume size | Subvolume # | Head volume | Neck volume | Head surface area | Neck surface area |
| Ideal | n/a | n/a | 0.115655 | 0.0136236 | 1.12779 | 0.336997 |
| Cubic 1 | 0.036 | 2768 | 0.115707 | 0.0134369 | 1.67962 | 0.373248 |
| Tetrahedral 1 | adaptive | 2900 | 0.115879 | 0.0134746 | 1.13330 | 0.339520 |
| Cubic 2 | 0.022 | 12164 | 0.115467 | 0.0140554 | 1.71336 | 0.464640 |
| Tetrahedral 2 | adaptive | 11885 | 0.115680 | 0.0134645 | 1.12921 | 0.336770 |
| | | | | | | |
| **Spine #3:** | Head radius: 0.254001 | Neck radius: 0.115128 | Neck length: 0.811319 | | | |
| Mesh | Subvolume size | Subvolume # | Head volume | Neck volume | Head surface area | Neck surface area |
| Ideal | n/a | n/a | 0.0680573 | 0.0337831 | 0.766707 | 0.586882 |
| Cubic 1 | 0.033 | 2680 | 0.0687115 | 0.0275996 | 1.18483 | 0.627264 |
| Tetrahedral 1 | adaptive | 2718 | 0.0677749 | 0.0338201 | 0.767768 | 0.594308 |
| Cubic 2 | 0.020 | 13016 | 0.0682880 | 0.0358400 | 1.16160 | 0.768000 |
| Tetrahedral 2 | adaptive | 12582 | 0.0680161 | 0.0336910 | 0.767281 | 0.588878 |
| | | | | | | |
| **Spine #4** | Head radius: 0.312020 | Neck radius: 0.0863628 | Neck length: 1.00114 | | | |
| Mesh | Subvolume size | Subvolume # | Head volume | Neck volume | Head surface area | Neck surface area |
| Ideal | n/a | n/a | 0.127099 | 0.0234585 | 1.19952 | 0.543254 |
| Cubic 1 | 0.039 | 2576 | 0.128129 | 0.0246767 | 1.80086 | 0.632736 |
| Tetrahedral 1 | adaptive | 2571 | 0.126159 | 0.0230094 | 1.19926 | 0.547544 |
| Cubic 2 | 0.021 | 16216 | 0.127061 | 0.0231155 | 1.80457 | 0.677376 |
| Tetrahedral 2 | adaptive | 16528 | 0.127319 | 0.0232958 | 1.20201 | 0.544621 |

| **Spine #5** | Head radius: 0.258623 | Neck radius: 0.0647370 | Neck length: 0.588977 | | | |
|---|---|---|---|---|---|---|
| Mesh | Subvolume size | Subvolume # | Head volume | Neck volume | Head surface area | Neck surface area |
| Ideal | n/a | n/a | 0.0724037 | 0.00775449 | 0.827130 | 0.239569 |
| Cubic 1 | 0.030 | 2992 | 0.0721440 | 0.00864000 | 1.23120 | 0.288000 |
| Tetrahedral 1 | adaptive | 2993 | 0.0721540 | 0.00769663 | 0.828733 | 0.241968 |
| Cubic 2 | 0.019 | 11504 | 0.0721018 | 0.00680413 | 1.24184 | 0.268584 |
| Tetrahedral 2 | adaptive | 11142 | 0.0724302 | 0.00764397 | 0.828225 | 0.239811 |
