# Supplementary material for: STEPS: efficient simulation of stochastic reaction–diffusion models in realistic morphologies
Source: BMC Syst Biol. 2012 May 10;6:36. doi: 10.1186/1752-0509-6-36 (PMC3472240; doi:10.1186/1752-0509-6-36)
Supplement: Additional file 4 — Validation. Detailed descriptions of the validation models used in this study, along with statistical analysis of results from STEPS simulations. [68]. [file 1752-0509-6-36-S4.pdf]

4. Validation

All the validations shown were run with STEPS version 1.3.0. Meshes were generated with CUBIT, version 12.0 [32].

First-order irreversible reaction:

$$A \xrightarrow{k} B \quad (17)$$

The mean and standard deviation of the number of molecules of reactant A is given by the analytical solutions to the master equation [17]:

$$N(t) = N_0 e^{-kt}, \ \sigma(t) = \sqrt{N_0 e^{-kt}(1 - e^{-kt})} \quad (18), (19)$$

The model is implemented as a Wmdirect solver object with k = 5/s, N_0 = 50 and volume = 1 μm^3.

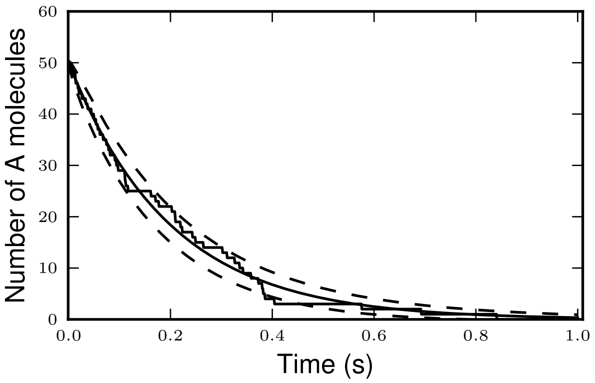

The figure above shows a single iteration of this model (thick line) compared to the analytical solution (broken lines: standard deviation of the stochastic simulation), while figure 6A shows the average of 1000 iterations.

First-order reversible reaction:

$$A \underset{k_b}{\overset{k_f}{\rightleftarrows}} B \quad (20)$$

The equilibrium concentrations can be computed from $d[A]/dt = d[B]/dt = 0$:

$$\frac{[A]_{eq}}{[A]_0} = \frac{\frac{k_b}{k_f}}{1 + \frac{k_b}{k_f}} \quad (21)$$

The model is implemented as a Wmdirect solver object with k_f = 10/s, k_b = 2/s, $[A]_0$ = 27.5 μM, $[B]_0$ = 0 μM and volume = 6 μm^3.

Figure 6B shows that the STEPS simulation evolves to the expected equilibrium concentrations, mean of 100 iterations shown.

Second order irreversible reaction:

$$A + B \xrightarrow{k} C \quad (22)$$

if $[A]_0 = [B]_0$ then:

$$\frac{1}{[A]} = kt + \frac{1}{[A]_0} \quad (23)$$

and a plot of 1/[A] vs time should be a straight line with slope k and intercept 1/[A0]. This is shown in figure 6C for a model implemented as a Wmdirect solver object with k = 50/μM.s, $[A]_0 = [B]_0$ = 20 μM and volume = 9 μm^3. Mean of 100 iterations is shown.

If $[A]_0 \neq [B]_0$ then:

$$\ln\left(\frac{[B]}{[A]}\right) = \ln\left(\frac{[B]_0}{[A]_0}\right) - \left([A]_0 - [B]_0\right)kt \quad (24)$$

Figure 6D shows the STEPS simulation compared to this analytical solution for a model implemented as a Wmdirect solver object with k = 5/μM.s, $[A]_0$ = 1 μM, $[B]_0$ = 0.5 μM and volume = 9 μm^3. Mean of 100 iterations is shown.

Production and degradation reactions

We consider simultaneous degradation and production:

$$A \xrightarrow{k_1} \varnothing, \quad \varnothing \xrightarrow{k_2} A \quad (25)$$

The stationary distribution found from the steady-state version of the master equation is given by (adapted from [38]):

$$\Phi(n) = \frac{1}{n!}\left(\frac{k_2}{k_1}\right)^n \exp\left[-\frac{k_2}{k_1}\right] \quad (26)$$

Figure 7 compares the stationary distribution from the STEPS simulation (histogram) with analytical solution (solid line) for a model implemented as a Wmdirect solver object with k_1 = 10/s, k_2 = 0.17μM/s, based on mean measurements of 100 iterations of simulations for 10,000 seconds and sampling the population of A every 0.1 seconds.

3D diffusion in infinite volume

The analytical solution for 3D diffusion from an instantaneous point source in an infinite volume is well known [47]:

$$C(r,t) = \frac{M}{8(\pi Dt)^{3/2}} \exp\left(\frac{-r^2}{4Dt}\right) \quad (27)$$

where *C* is concentration, *M* is the number or amount of molecules, *D* is the diffusion constant and *r* is the radial distance from source.

To approximate this type of diffusion we created a tetrahedral mesh of sphere radius 10 μm in CUBIT with 32,552 voxels. This was simulated with the 'Tetexact' solver in STEPS using $D = 20$ μm^2/s (Figure 8A). The initial condition was 5,000,000 molecules into the center tetrahedron. This simulation was run to 0.2 seconds, ensuring no boundary events occurred. Data in Figure 8A are mean of 10 iterations, sampled concentration in 10000 voxels selected at random, but with a bias towards central voxels. We spatially averaged spherical shells with a width 0.33 μm at the indicated radius.

The small deviation from the analytical solution in the first time bins is due to the initial concentration not being into an infinitely small volume. This is supported by the fact that the deviation depends on the size of this central voxel (not shown).

1D diffusion in a finite tube from a point source at one end

The analytical solution for 1D diffusion from the boundary ($x = 0$) in a finite tube was found by applying our boundary conditions to the general solution in [47]:

$$C = C_0\left[1 + 2\sum_{n=1}^{\infty}\cos\left(\frac{n\pi x}{L}\right)\exp\left\{-\left(\frac{n\pi}{L}\right)^2 Dt\right\}\right] \quad (28)$$

The number of significant terms in the sum (from 1 to infinity) depends on the size of the exponential term, and therefore the time, but except for very early times only the first few terms are significant. It was found that for the times plotted fewer than 10 terms were significant, and 100 terms are summed to ensure accuracy.

To approximate this type of diffusion we created a tetrahedral mesh of a cylinder, radius 0.2 μm, length 10 μm (in CUBIT) with 12,033 voxels. This was simulated with the Tetexact solver in STEPS using $D = 200$ μm^2/s. As initial condition we injected 250,000 molecules uniformly over all tetrahedra with a face on the $x = 0$ boundary. This simulation was run to 0.15 seconds, when the concentration gradient had become close to zero everywhere. Data in Figure 8B are mean of 10 iterations, with the concentration sampled in all voxels in 0.5 μm bins (each bin containing about 600 voxels).

1D diffusion in a semi-infinite tube with clamped concentration

The analytical solution for 1D diffusion in a semi-infinite tube with a clamped concentration at the boundary ($x = 0$) is [47]:

$$C(x,t) = C_0\left[1 - erf\left(\frac{x}{2\sqrt{Dt}}\right)\right] \quad (29)$$

where erf is the error-function.

This was simulated with the same mesh as the previous simulation, using the Tetexact solver in STEPS with $D = 100$ μm^2/s. The concentration in all tetrahedra with a face on the $x = 0$ boundary was clamped to 1 mM using the setTetClamped() method, initial condition was zero concentration elsewhere. This simulation was run to 0.05 seconds, ensuring no boundary events. Data in Figure 8C are mean of 10 iterations, with the concentration sampled in all voxels in 0.33 μm bins.

1D diffusion in a finite tube with constant influx at both ends

The analytical solution for 1D diffusion in a finite tube with a constant and equal influx of the same species of molecules at both ends is [68]:

$$C(x,t) = \frac{JL}{D}\left\{\frac{Dt}{L^2} + \frac{3x^2 - L^2}{6L^2} - \frac{2}{\pi^2}\sum_{n=1}^{\infty}\frac{(-1)^n}{n^2}\exp\left[-D\left(\frac{n\pi}{L}\right)^2 t\right]\cos\left(\frac{n\pi x}{L}\right)\right\} \quad (30)$$

where the tube has a dimension from $-L$ to L and J is the diffusion flux (number of molecules per m^{2}s). Again, only the first few terms are significant in the sum.

This was simulated with the same mesh as the previous simulations, using the Tetexact solver in STEPS with $D = 50$ μm^2/s. Initial condition was zero concentration everywhere. To simulate the constant influx a reaction scheme was set up in all 50 tetrahedra with a face on the $x = \pm$L boundaries. This reaction A -> X created a constant source of X molecules because the the number of A was clamped to 1 in each tetrahedron; the reaction rate was set at 12000/s to generate a total flux over the border of the tube of 300,000/s. This simulation was run to 0.15 seconds. Data in Figure 8D are mean of 30 iterations, with the concentration sampled in all voxels in 0.5 μm bins.

Lack of effect of diffusion on a first-order irreversible reaction

The reaction of equation 17, with k = 1/s and $[A]_0$ = 40 nM (uniform distribution), was implemented in a tetrahedral mesh of a sphere with a 1 μm radius (volume = 4.1 μm^3, 9457 voxels) and run using the Tetexact solver with D = 1 μm^2/s. Data in Figure 9A are mean of 1000 iterations.

Lack of effect of slow diffusion on discrete production-degradation system

To investigate the effect of diffusion and subvolume size on a very discrete reaction-diffusion problem we implemented a model of simultaneous degradation and production, differing from (25) in that the degradation reaction is second-order:

$$A + B \xrightarrow{k_1} B, \quad \varnothing \xrightarrow{k_2} A \tag{31}$$

The stationary distribution found from the steady-state version of the master equation is given by [38]:

$$\Phi(n) = \frac{1}{n!}\left(\frac{k_2 v^2}{k_1 B_0}\right)^n \exp\left[-\frac{k_2 v^2}{k_1 B_0}\right] \tag{32}$$

The diffusion coefficient of molecules A and B was set to the low value D = 10 μm^2/s and the reaction constant for the second-order reaction was set to the value of k_1 = 100/ μM.s and the production rate was set to k2 = 4nM/s. With the initial injection of just 1 B molecule these reaction parameters are expected to keep the mean number of molecules in the system at fewer than 10. The lower bound for acceptable tetrahedron size for the well-mixed condition was estimated as 0.2μm (see Subvolume Size) and the model was simulated in a uniform mesh of 73 tetrahedrons representing a total volume of 1μm^3, corresponding to a tetrahedron size of approximately 0.5μm.

Figure 9B compares the stationary distribution from the STEPS simulation (histogram) with analytical solution (white circles) for a model implemented as a Tetexact solver object with based on a simulation for 300,000 seconds and sampling the population of A every second.

Degradation-diffusion process with initially separated reactants

$$A + B \xrightarrow{k} \varnothing \tag{33}$$

Though this is a reaction-diffusion model, the assumption for the analytical approximation is that the reaction zone is much smaller than the diffusion length. This gives the boundary condition (at x = 0) that the concentration is clamped to zero. The solution then doesn't include any reaction term [48]:

$$N_A = -\frac{4N_{A0}}{\pi}\sum_{n=0}^{\infty}\frac{1}{2n+1}\exp\left[-\frac{D(2n+1)^2\pi^2 t}{4L^2}\right].\sin\left[\frac{(2n+1)\pi x}{2L}\right] \tag{34}$$

Except at very early times, only the first few terms are significant (for the plotted time-points at most 5 terms were significant), for Figure 10 the first 100 terms were computed. The reaction rate was set very high, k = 100,000/μM.s, to enforce the boundary condition. Simulation using the Tetexact solver on a square cuboid of 40 μm by 4 μm by 4 μm (11713 voxels) was run to 0.4 seconds. Reactants A and B were uniformly distributed initially in different 20 μm halves of this square cuboid, 1,000,000 molecules of each, with D_A = D_B = 400 μm^2/s. Data in Figure 10 are mean and SD of 10 iterations.

4.1 Statistical analysis

Below is a table of statistical analysis of the reaction validation models. In the first 5 models the output from STEPS at every time-point (except where equilibrium must first be reached in the first-order reversible reaction model) is analyzed and in the last model output from molecule number 3-18 is analyzed. In each case the output is converted to a 95% confidence interval, assuming a normal distribution in each case, then compared to the analytical mean value, with a summary of whether the true mean fell within the confidence interval of the STEPS output:

		**First order irreversible reaction**		
Time	95% confidence interval lower-bound	95% confidence interval upper-bound	Analytical mean	Analytical mean fell within confidence interval?
0.05	38.744684097	39.1173159031	38.940039154	YES
0.1	30.134684232	30.579315768	30.326532986	YES
0.15	23.355250752	23.812749248	23.618327637	YES
0.2	18.091665084	18.5263349162	18.393972059	YES
0.25	14.119285808	14.5167141918	14.325239843	YES
0.3	10.902995444	11.2710045557	11.156508007	YES
0.35	8.4908882166	8.82711178338	8.6886971725	YES

0.4	6.6697753747	6.97222462529	6.7667641618	YES
0.45	5.1707725514	5.43922744861	5.2699612281	YES
0.5	3.9865402269	4.22545977307	4.1042499312	YES
0.55	3.0837803144	3.29821968565	3.1963930603	YES
0.6	2.3854203342	2.57257966582	2.4893534184	YES
0.65	1.8237929174	1.98820708256	1.9387103916	YES
0.7	1.3880711681	1.53392883193	1.5098691711	YES
0.75	1.0945365863	1.22346341374	1.1758872928	YES
0.8	0.8573955033	0.9726044967	0.9157819444	YES
0.85	0.679300744	0.78069925601	0.7132116955	YES
0.9	0.5234014941	0.61459850592	0.5554498269	YES
0.95	0.4183415019	0.49965849813	0.4325847602	YES
1.0	0.3214190624	0.39258093762	0.33689735	YES
TOTAL:<br>YES 20, NO 0				
		**First order reversible reaction**		
Time	95% confidence interval lower-bound	95% confidence interval upper-bound	Analytical mean	Analytical mean fell within confidence interval?
0.7	4.6036826842	4.61500361428	4.6126079531	YES
0.7	23.060644105	23.0719650346	23.063039766	YES
0.75	4.6043764498	4.61783019099	4.6126079531	YES
0.75	23.057817528	23.0712712689	23.063039766	YES
0.8	4.6056777058	4.61746437197	4.6126079531	YES

0.8	23.058183347	23.069970013	23.063039766	YES
0.85	4.6055743972	4.61749018869	4.6126079531	YES
0.85	23.05815753	23.0700733216	23.063039766	YES
0.9	4.6076281608	4.62048999841	4.6126079531	YES
0.9	23.05515772	23.068019558	23.063039766	YES
0.95	4.6025742023	4.61540913469	4.6126079531	YES
0.95	23.060238584	23.0730735165	23.063039766	YES
1.0	4.6015430629	4.6149015081	4.6126079531	YES
1.0	23.060746211	23.0741046559	23.063039766	YES
TOTAL:<br>YES 14, NO 0				
		**Second order irreversible reaction, equal reactant concentrations**		
Time	95% confidence interval lower-bound	95% confidence interval upper-bound	Analytical mean	Analytical mean fell within confidence interval?
0.05	2.539519881	2.5523868382	2.55	YES
0.05	2.5395680842	2.55255391225	2.55	YES
0.1	5.043140848	5.08198146778	5.05	YES
0.1	5.0434765087	5.08249705507	5.05	YES
0.15	7.5427551473	7.59586099149	7.55	YES
0.15	7.5436830851	7.59683608527	7.55	YES

0.2	10.02709062	10.1179681989	10.05	YES
0.2	10.028538974	10.1198898363	10.05	YES
0.25	12.47888696	12.5988567069	12.55	YES
0.25	12.481374448	12.6015918148	12.55	YES
0.3	14.948654619	15.1169544679	15.05	YES
0.3	14.952430006	15.1206860992	15.05	YES
0.35	17.308230602	17.5242073801	17.55	NO
0.35	17.312529629	17.5299849158	17.55	NO
0.4	19.856894394	20.127814938	20.05	YES
0.4	19.862843202	20.1351446952	20.05	YES
0.45	22.24392394	22.6063168648	22.55	YES
0.45	22.251207929	22.6157403324	22.55	YES
0.5	24.706206074	25.1322063397	25.05	YES
0.5	24.715126337	25.1439174051	25.05	YES
0.55	27.188368771	27.6969714823	27.55	YES
0.55	27.200659889	27.7097028076	27.55	YES
0.6	29.673740905	30.2414409438	30.05	YES
0.6	29.689993412	30.2550085232	30.05	YES
0.65	32.175337911	32.7650808522	32.55	YES
0.65	32.194200561	32.7812516956	32.55	YES
0.7	34.667617847	35.2894954158	35.05	YES
0.7	34.690902547	35.3068676976	35.05	YES
0.75	37.119640546	37.8240451564	37.55	YES
0.75	37.147340414	37.8430068706	37.55	YES
0.8	39.480622752	40.241777042	40.05	YES

0.8	39.513904099	40.2612997576	40.05	YES
0.85	42.056050498	42.9224278653	42.55	YES
0.85	42.091177559	42.9472997293	42.55	YES
0.9	44.604884118	45.6069559282	45.05	YES
0.9	44.639618606	45.6398408762	45.05	YES
0.95	47.114528389	48.2144198127	47.55	YES
0.95	47.154310926	48.2501486764	47.55	YES
1.0	49.55734391	50.6724730108	50.05	YES
1.0	49.603597641	50.7096977505	50.05	YES
TOTAL:<br>YES 38, NO 2				
		**Second order irreversible reaction, unequal reactant concentrations**		
Time	95% confidence interval lower-bound	95% confidence interval upper-bound	Analytical mean	Analytical mean fell within confidence interval?
0.05	-0.819968301	-0.8177259198	-0.818147181	YES
0.1	-0.9471777392	-0.9438354641	-0.943147181	NO
0.15	-1.0712144417	-1.06704414112	-1.068147181	YES
0.2	-1.1965678408	-1.19089961611	-1.193147181	YES
0.25	-1.3215196978	-1.3144182997	-1.318147181	YES
0.3	-1.4466412619	-1.4389451409	-1.443147181	YES
0.35	-1.5719361037	-1.5636269921	-1.568147181	YES

0.4	-1.69917606	-1.6894532048	-1.693147181	YES
0.45	-1.8229894905	-1.81190076831	-1.818147181	YES
0.5	-1.9481134542	-1.9357071361	-1.943147181	YES
0.55	-2.0750802832	-2.0615550592	-2.068147181	YES
0.6	-2.2033233424	-2.188294371	-2.193147181	YES
0.65	-2.3304321809	-2.3135180564	-2.318147181	YES
0.7	-2.4577523544	-2.4388364304	-2.443147181	YES
0.75	-2.5883975157	-2.5678899036	-2.568147181	YES
0.8	-2.7100621734	-2.6871569793	-2.693147181	YES
0.85	-2.836923514	-2.812745443	-2.818147181	YES
0.9	-2.9646697549	-2.9383024571	-2.943147181	YES
0.95	-3.0842700765	-3.0546526957	-3.068147181	YES
1.0	-3.2082728617	-3.1754686224	-3.193147181	YES
TOTAL:<br>YES 19, NO 1				
		**Production-degradation**		
Molecule number	95% confidence interval lower-bound	95% confidence interval upper-bound	Analytical mean	Analytical mean fell within confidence interval?
3	0.0075097101	0.00761646478	0.007566655	YES
4	0.0188291642	0.01900308977	0.0189166374	YES
5	0.0377693963	0.03803805307	0.0378332748	YES
6	0.0628159356	0.06312935028	0.063055458	YES
7	0.0898295042	0.09021794278	0.0900792257	YES

8	0.1125394679	0.11295717382	0.1125990321	YES
9	0.1248865409	0.12528613006	0.1251100357	YES
10	0.1249197956	0.1253189407	0.1251100357	YES
11	0.11334011298	0.11376472081	0.1137363961	YES
12	0.0946421873	0.09505301095	0.0947803301	YES
13	0.0728228553	0.07316867636	0.0729079462	YES
14	0.051939112	0.0522077934	0.0520771044	YES
15	0.0345789859	0.03482952857	0.0347180696	YES
16	0.0215999637	0.02179699933	0.0216987935	YES
17	0.0126535429	0.01279184802	0.0127639962	YES
18	0.0070919661	0.00719978271	0.007091109	NO
TOTAL: YES 15, NO 1				
		**First order irreversible reaction-diffusion**		
Time	95% confidence interval lower-bound	95% confidence interval upper-bound	Analytical mean	Analytical mean fell within confidence interval?
0.1	36.108362959	36.255082315	36.176022112	YES
0.2	32.624546949	32.8070467145	32.733418443	YES
0.3	29.41207441	29.6234088559	29.618421827	YES
0.4	26.632749961	26.8590111661	26.799856333	YES
0.5	24.052918916	24.2837323567	24.249512808	YES
0.6	21.825357739	22.0670403028	21.941866558	YES

0.7	19.770319637	20.014462564	19.853821883	YES
0.8	17.851727617	18.0997062596	17.964480931	YES
0.9	16.152458536	16.3910215326	16.254934542	YES
1.0	14.641077975	14.8718659756	14.708073001	YES
1.1	13.263547345	13.4851318668	13.308414799	YES
1.2	11.9830111675	12.2029059712	12.041951684	YES
1.3	10.840727898	11.0582939116	10.89600847	YES
1.4	9.8184304718	10.0327805206	9.8591161711	YES
1.5	8.859773598	9.06596748093	8.9208972204	YES
1.6	7.9831987698	8.18339205609	8.0719616074	YES
1.7	7.2198227065	7.41310894419	7.3038128994	YES
1.8	6.5206763632	6.70493508496	6.6087632057	YES
1.9	5.8880501516	6.06617543188	5.9798562354	YES
2.0	5.3517201552	5.52222725008	5.4107976763	YES
2.1	4.8349779693	4.99867194545	4.8958921989	YES
2.2	4.3512639524	4.50925602688	4.4299864563	YES
2.3	3.9396401839	4.09008110765	4.008417507	YES
2.4	3.564965386	3.70832129677	3.6269661475	YES
2.5	3.2521390223	3.38945279689	3.2818146842	YES
2.6	2.9342166583	3.06528531851	2.9695087253	YES
2.7	2.651587021	2.7769907373	2.6869226078	YES
2.8	2.3990640385	2.51856053266	2.4312281149	YES
2.9	2.186575462	2.30245613838	2.1998661702	YES
3.0	1.9823757801	2.09165628333	1.9905212255	YES
TOTAL:<br>YES 30, NO 0				
