# Supplementary material for: STEPS: efficient simulation of stochastic reaction–diffusion models in realistic morphologies
Source: BMC Syst Biol. 2012 May 10;6:36. doi: 10.1186/1752-0509-6-36 (PMC3472240; doi:10.1186/1752-0509-6-36)
Supplement: Additional file 5 — Simulator Efficiency Test Model. A description of the model used to study efficiency of STEPS, MesoRD and Smoldyn, along with model files for each simulator. [file 1752-0509-6-36-S5.pdf]

5. Simulator Efficiency Test Model

We designed a test model of several chemical species and reactions instead of using previous test models that often only include only a very small number of diffusing species and reactions. The typical problems that reaction-diffusion simulators are applied to will of course involve even larger systems, but the test model here is an improvement on previous simulator comparisons. We ran the model in STEPS 1.3.0, MesoRD 0.3 [19] and Smoldyn 2.10 [12].

We started with 10 chemical species, named ‘A’, ‘B’, ‘C’, ‘D’, ‘E’, ‘F’, ‘G’, ‘H’, ‘I’ and ‘J’.

The diffusion coefficients were set to a different value for each species, covering a broad range:
$D_A = 100\mu m^2/s$
$D_B = 90\mu m^2/s$
$D_C = 80\mu m^2/s$
$D_D = 70\mu m^2/s$
$D_E = 60\mu m^2/s$
$D_F = 50\mu m^2/s$
$D_G = 40\mu m^2/s$
$D_H = 30\mu m^2/s$
$D_I = 20\mu m^2/s$
$D_J = 10\mu m^2/s$

The interactions of the species were described by 8 reaction channels (6 of which form 3 reversible reactions) with various rate constants:

$$A + B \xrightarrow{1000/\mu M.s} C$$
$$C \xrightarrow{100/s} A + B$$
$$C + D \xrightarrow{100/\mu M.s} E$$
$$E \xrightarrow{10/s} C + D$$
$$F + G \xrightarrow{10/\mu M.s} H$$
$$H \xrightarrow{1/s} F + G$$
$$H + I \xrightarrow{1/\mu M.s} J$$
$$J \xrightarrow{0.1/s} A$$

The simulation geometry was a simple cubic volume of side length $3\mu m$, giving a total volume of $27\mu m^3$. It’s possible to set simple boundaries such as these in the Smoldyn configuration file. In STEPS different tetrahedral meshes were generated in CUBIT for different simulations to test a range of subvolume sizes, though in all cases the boundaries were the same.

Two different models were tested with a different total number of molecules in the system. In both cases the starting number of molecules was set to a different number for each distinct species:

Model 1:
$N_A = 100$
$N_B = 200$
$N_C = 300$
$N_D = 400$
$N_E = 500$
$N_F = 600$
$N_G = 700$
$N_H = 800$
$N_I = 900$
$N_J = 1000$

Model 2:
$N_A = 1000$
$N_B = 2000$
$N_C = 3000$
$N_D = 4000$
$N_E = 5000$
$N_F = 6000$
$N_G = 7000$
$N_H = 8000$
$N_I = 9000$
$N_J = 10000$

In all simulations all molecules were injected uniformly throughout the volume and the simulation was run to 1s. The figure below shows the total number of molecules in the system in a STEPS simulation of Model 1 over time.

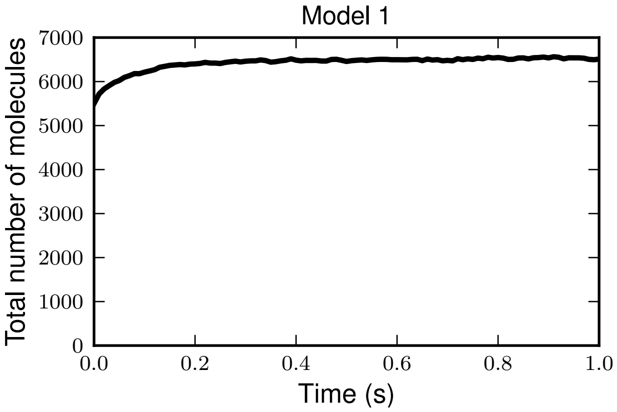

The configuration scripts are given here for the lower molecule number case, with a 454 tetrahedron mesh import in STEPS and 0.1ms time-step in Smoldyn (for MesoRD the cube size is specified from command line). The meshes for the STEPS simulations are

provided in separate files which may be downloaded from the STEPS website. The example STEPS script contains commands to plot the collected data with Matplotlib:

STEPS:

```
# model1.py
# Software efficiency test model in STEPS
# Model 1: lower molecule numbers

########################################################################

import steps.model as smod
import steps.geom as stetmesh
import steps.rng as srng
import steps.solver as ssolv
import steps.utilities.meshio as meshio

import time
from pylab import *

########################################################################

# The initial molecule counts
N0A = 100
N0B = 200
N0C = 300
N0D = 400
N0E = 500
N0F = 600
N0G = 700
N0H = 800
N0I = 900
N0J = 1000

mdl  = smod.Model()

# The chemical species
A = smod.Spec('A', mdl)
B = smod.Spec('B', mdl)
C = smod.Spec('C', mdl)
D = smod.Spec('D', mdl)
E = smod.Spec('E', mdl)
F = smod.Spec('F', mdl)
G = smod.Spec('G', mdl)
H = smod.Spec('H', mdl)
I = smod.Spec('I', mdl)
J = smod.Spec('J', mdl)
```

```
volsys = smod.Volsys('vsys',mdl)

# The reactions
R1 = smod.Reac('R1', volsys, lhs = [A, B], rhs = [C],  kcst = 1000.0e6)
R2 = smod.Reac('R2', volsys, lhs = [C],  rhs = [A,B], kcst = 100)
R3 = smod.Reac('R3', volsys, lhs = [C, D], rhs = [E], kcst = 100e6)
R4 = smod.Reac('R4', volsys, lhs = [E], rhs = [C,D], kcst = 10)
R5 = smod.Reac('R5', volsys, lhs = [F, G], rhs = [H], kcst = 10e6)
R6 = smod.Reac('R6', volsys, lhs = [H], rhs = [F,G], kcst = 1)
R7 = smod.Reac('R7', volsys, lhs = [H, I], rhs = [J],  kcst = 1e6)
R8 = smod.Reac('R8', volsys, lhs = [J],  rhs = [A], kcst = 0.1)

# The diffusion rules
D1 = smod.Diff('D1', volsys, A, dcst = 100e-12)
D2 = smod.Diff('D2', volsys, B, dcst = 90e-12)
D3 = smod.Diff('D3', volsys, C, dcst = 80e-12)
D4 = smod.Diff('D4', volsys, D, dcst = 70e-12)
D5 = smod.Diff('D5', volsys, E, dcst = 60e-12)
D6 = smod.Diff('D6', volsys, F, dcst = 50e-12)
D7 = smod.Diff('D7', volsys, G, dcst = 40e-12)
D8 = smod.Diff('D8', volsys, H, dcst = 30e-12)
D9 = smod.Diff('D9', volsys, I, dcst = 20e-12)
D10 = smod.Diff('D10', volsys, J, dcst = 10e-12)

# Import mesh from Abaqus file
mesh = meshio.importAbaqus('cube_3_3_3_454tets.inp', 1e-6)[0]

# Create compartment of whole mesh
comp1 = stetmesh.TmComp('comp1', mesh, range(mesh.ntets))
comp1.addVolsys('vsys')

# Create random number generator
rng = srng.create('mt19937', 256)
rng.initialize(int(time.time()%4294967295))

# Create solver object
sim = ssolv.Tetexact(mdl, mesh, rng)
sim.reset()

# Create data structures
tpnts = arange(0.0, 1.01, 0.01)
ntpnts = tpnts.shape[0]
res = zeros([ntpnts, 10])

# Set initial conditions
```

```
sim.setCompCount('comp1', 'A', N0A)
sim.setCompCount('comp1', 'B', N0B)
sim.setCompCount('comp1', 'C', N0C)
sim.setCompCount('comp1', 'D', N0D)
sim.setCompCount('comp1', 'E', N0E)
sim.setCompCount('comp1', 'F', N0F)
sim.setCompCount('comp1', 'G', N0G)
sim.setCompCount('comp1', 'H', N0H)
sim.setCompCount('comp1', 'I', N0I)
sim.setCompCount('comp1', 'J', N0J)

# Run simulation and collect data
specs = ['A','B','C','D','E','F','G','H','I','J']
for t in range(0, ntpnts):
    sim.run(tpnts[t])
    for s in range(10):
        res[t, s] = sim.getCompCount('comp1', specs[s])

# Plot results
for s in range(10):
    plot(tpnts, res[:,s], label = specs[s])
xlabel('Time (s)' )
ylabel('Number of molecules')
legend()
show()

########################################################################
## END
```

SMOLDYN:

```
# Software efficiency test model in Smoldyn
# Model 1: lower molecule numbers
# units are nm, us

graphics none

dim 3
max_species 10
species A
species B
species C
species D
species E
species F
species G
```

```
species H
species I
species J

max_mol 10000

difc A 100
difc B 90
difc C 80
difc D 70
difc E 60
difc F 50
difc G 40
difc H 30
difc I 20
difc J 10

time_start 0.0
time_stop 1000000
time_step 100.0

boundaries 0 0 3000 r
boundaries 1 0 3000 r
boundaries 2 0 3000 r

mol 100 A u u u
mol 200 B u u u
mol 300 C u u u
mol 400 D u u u
mol 500 E u u u
mol 600 F u u u
mol 700 G u u u
mol 800 H u u u
mol 900 I u u u
mol 1000 J u u u

output_files model1.txt
cmd e molcount model1.txt

reaction one  A + B -> C  1660e0
reaction two  C -> A + B  100e-6
reaction three  C + D -> E  166e0
reaction four E -> C + D 10e-6
reaction five F + G -> H 16.6e0
reaction six H -> F + G 1e-6
reaction seven H + I -> J 1.66e0
```

reaction eight J -> A 0.1e-6

end_file

MESORD:


```
<?xml version="1.0" encoding="UTF-8"?>
<sbml xmlns="http://www.sbml.org/sbml/level2/version4" level="2" version="4">
<model name="STEPS Efficiency test model">


  <!-- ********************
       * UNIT DEFINITIONS *
       ******************** -->
  <listOfUnitDefinitions>
    <unitDefinition id="um">
      <listOfUnits>
        <unit kind="metre" scale="-6"/>
      </listOfUnits>
    </unitDefinition>
    <unitDefinition id="pMps">
      <listOfUnits>
        <unit kind="mole"   exponent="-1"/>
        <unit kind="litre"  exponent="+1"/>
        <unit kind="second" exponent="-1"/>
      </listOfUnits>
    </unitDefinition>
    <unitDefinition id="um2ps">
      <listOfUnits>
        <unit kind="meter"   scale="-12"/>
        <unit kind="second" exponent="-1"/>
      </listOfUnits>
    </unitDefinition>
    <unitDefinition id="ps">
        <listOfUnits>
            <unit kind="second" exponent="-1"/>
        </listOfUnits>
    </unitDefinition>
  </listOfUnitDefinitions>


  <!-- ****************
       * COMPARTMENTS *
       **************** -->
  <listOfCompartments>
    <compartment id="CompartmentOne" units="litre">
     <annotation>
         <MesoRD:csg xmlns:MesoRD="http://www.icm.uu.se">
             <MesoRD:scale MesoRD:x="1.0"
                           MesoRD:y="1.0"
                           MesoRD:z="1.0"
                           MesoRD:units="um">
                    <MesoRD:box MesoRD:x="3.0"
                                MesoRD:y="3.0"
                                MesoRD:z="3.0"
                                MesoRD:units="um"/>
             </MesoRD:scale>
         </MesoRD:csg>
     </annotation>
    </compartment>
  </listOfCompartments>
```

```
<!-- ***********
     * SPECIES *
     *********** -->
<listOfSpecies>
  <species id="A"
           boundaryCondition="false"
           compartment="CompartmentOne"
            hasOnlySubstanceUnits="true"
           initialAmount="100"
           name="A"
           substanceUnits="item">
    <annotation>
        <MesoRD:species_diffusion xmlns:MesoRD="http://www.icm.uu.se">
            <MesoRD:diffusion MesoRD:compartment="CompartmentOne"
                              MesoRD:rate="100e-8"
                              MesoRD:units="cm2ps"/>
        </MesoRD:species_diffusion>
    </annotation>
  </species>
  <species id="B"
           boundaryCondition="false"
           compartment="CompartmentOne"
            hasOnlySubstanceUnits="true"
           initialAmount="200"
           name="B"
           substanceUnits="item">
    <annotation>
        <MesoRD:species_diffusion xmlns:MesoRD="http://www.icm.uu.se">
            <MesoRD:diffusion MesoRD:compartment="CompartmentOne"
                              MesoRD:rate="90e-8"
                              MesoRD:units="cm2ps"/>
        </MesoRD:species_diffusion>
    </annotation>
  </species>
  <species id="C"
           boundaryCondition="false"
           compartment="CompartmentOne"
            hasOnlySubstanceUnits="true"
           initialAmount="300"
           name="C"
           substanceUnits="item">
    <annotation>
        <MesoRD:species_diffusion xmlns:MesoRD="http://www.icm.uu.se">
            <MesoRD:diffusion MesoRD:compartment="CompartmentOne"
                              MesoRD:rate="80e-8"
                              MesoRD:units="cm2ps"/>
        </MesoRD:species_diffusion>
    </annotation>
  </species>
  <species id="D"
           boundaryCondition="false"
           compartment="CompartmentOne"
            hasOnlySubstanceUnits="true"
           initialAmount="400"
           name="D"
           substanceUnits="item">
    <annotation>
        <MesoRD:species_diffusion xmlns:MesoRD="http://www.icm.uu.se">
            <MesoRD:diffusion MesoRD:compartment="CompartmentOne"
                              MesoRD:rate="70e-8"
                              MesoRD:units="cm2ps"/>
        </MesoRD:species_diffusion>
    </annotation>
```

```
</species>
<species id="E"
         boundaryCondition="false"
         compartment="CompartmentOne"
          hasOnlySubstanceUnits="true"
         initialAmount="500"
         name="E"
         substanceUnits="item">
  <annotation>
      <MesoRD:species_diffusion xmlns:MesoRD="http://www.icm.uu.se">
          <MesoRD:diffusion MesoRD:compartment="CompartmentOne"
                            MesoRD:rate="60e-8"
                            MesoRD:units="cm2ps"/>
      </MesoRD:species_diffusion>
  </annotation>
</species>
<species id="F"
         boundaryCondition="false"
         compartment="CompartmentOne"
          hasOnlySubstanceUnits="true"
         initialAmount="600"
         name="F"
         substanceUnits="item">
  <annotation>
      <MesoRD:species_diffusion xmlns:MesoRD="http://www.icm.uu.se">
          <MesoRD:diffusion MesoRD:compartment="CompartmentOne"
                            MesoRD:rate="50e-8"
                            MesoRD:units="cm2ps"/>
      </MesoRD:species_diffusion>
  </annotation>
</species>
<species id="G"
         boundaryCondition="false"
         compartment="CompartmentOne"
          hasOnlySubstanceUnits="true"
         initialAmount="700"
         name="G"
         substanceUnits="item">
  <annotation>
      <MesoRD:species_diffusion xmlns:MesoRD="http://www.icm.uu.se">
          <MesoRD:diffusion MesoRD:compartment="CompartmentOne"
                            MesoRD:rate="40e-8"
                            MesoRD:units="cm2ps"/>
      </MesoRD:species_diffusion>
  </annotation>
</species>
<species id="H"
         boundaryCondition="false"
         compartment="CompartmentOne"
          hasOnlySubstanceUnits="true"
         initialAmount="800"
         name="H"
         substanceUnits="item">
  <annotation>
      <MesoRD:species_diffusion xmlns:MesoRD="http://www.icm.uu.se">
          <MesoRD:diffusion MesoRD:compartment="CompartmentOne"
                            MesoRD:rate="30e-8"
                            MesoRD:units="cm2ps"/>
      </MesoRD:species_diffusion>
  </annotation>
</species>
<species id="I"
         boundaryCondition="false"
         compartment="CompartmentOne"
```

```
             hasOnlySubstanceUnits="true"
            initialAmount="900"
            name="I"
            substanceUnits="item">
    <annotation>
        <MesoRD:species_diffusion xmlns:MesoRD="http://www.icm.uu.se">
            <MesoRD:diffusion MesoRD:compartment="CompartmentOne"
                              MesoRD:rate="20e-8"
                              MesoRD:units="cm2ps"/>
        </MesoRD:species_diffusion>
    </annotation>
  </species>
  <species id="J"
            boundaryCondition="false"
            compartment="CompartmentOne"
             hasOnlySubstanceUnits="true"
            initialAmount="1000"
            name="J"
            substanceUnits="item">
    <annotation>
        <MesoRD:species_diffusion xmlns:MesoRD="http://www.icm.uu.se">
            <MesoRD:diffusion MesoRD:compartment="CompartmentOne"
                              MesoRD:rate="10e-8"
                              MesoRD:units="cm2ps"/>
        </MesoRD:species_diffusion>
    </annotation>
  </species>
</listOfSpecies>


<!-- **************
     * PARAMETERS *
     ************** -->
<listOfParameters>
  <parameter id="k1" units="pMps" value="1000.0e6"/>
  <parameter id="k2" units="ps" value="100"/>
  <parameter id="k3" units="pMps" value="100.0e6"/>
  <parameter id="k4" units="ps" value="10"/>
  <parameter id="k5" units="pMps" value="10.0e6"/>
  <parameter id="k6" units="ps" value="1"/>
  <parameter id="k7" units="pMps" value="1.0e6"/>
  <parameter id="k8" units="ps" value="0.1"/>
</listOfParameters>



<!-- *************
     * REACTIONS *
     ************* -->
<listOfReactions>
  <reaction id="Reaction1" reversible="false">
    <listOfReactants>
      <speciesReference species="A"/>
      <speciesReference species="B"/>
    </listOfReactants>
    <listOfProducts>
      <speciesReference species="C"/>
    </listOfProducts>
    <kineticLaw>
      <math xmlns:MesoRD="http://www.w3.org/1998/Math/MathML">
     <apply>
       <times/>
       <ci>k1</ci>
       <ci>A</ci>
```

```
      <ci>B</ci>
    </apply>
  </math>
   </kineticLaw>
  <!-- <annotation xmlns:MesoRD="http://www.icm.uu.se">
     <MesoRD:location MesoRD:compartment="CompartmentOne"/>
   </annotation> -->
</reaction>
<reaction id="Reaction2" reversible="false">
  <listOfReactants>
    <speciesReference species="C"/>
  </listOfReactants>
  <listOfProducts>
    <speciesReference species="A"/>
    <speciesReference species="B"/>
  </listOfProducts>
  <kineticLaw>
    <math xmlns:MesoRD="http://www.w3.org/1998/Math/MathML">
    <apply>
      <times/>
      <ci>k2</ci>
      <ci>C</ci>
    </apply>
  </math>
   </kineticLaw>
  <!-- <annotation xmlns:MesoRD="http://www.icm.uu.se">
     <MesoRD:location MesoRD:compartment="CompartmentOne"/>
   </annotation> -->
</reaction>
<reaction id="Reaction3" reversible="false">
  <listOfReactants>
    <speciesReference species="C"/>
    <speciesReference species="D"/>
  </listOfReactants>
  <listOfProducts>
    <speciesReference species="E"/>
  </listOfProducts>
  <kineticLaw>
    <math xmlns:MesoRD="http://www.w3.org/1998/Math/MathML">
    <apply>
      <times/>
      <ci>k3</ci>
      <ci>C</ci>
      <ci>D</ci>
    </apply>
  </math>
   </kineticLaw>
  <!-- <annotation xmlns:MesoRD="http://www.icm.uu.se">
     <MesoRD:location MesoRD:compartment="CompartmentOne"/>
   </annotation> -->
</reaction>
<reaction id="Reaction4" reversible="false">
  <listOfReactants>
    <speciesReference species="E"/>
  </listOfReactants>
  <listOfProducts>
    <speciesReference species="C"/>
    <speciesReference species="D"/>
  </listOfProducts>
  <kineticLaw>
    <math xmlns:MesoRD="http://www.w3.org/1998/Math/MathML">
    <apply>
      <times/>
      <ci>k4</ci>
```

```
      <ci>E</ci>
    </apply>
  </math>
  </kineticLaw>
 <!-- <annotation xmlns:MesoRD="http://www.icm.uu.se">
    <MesoRD:location MesoRD:compartment="CompartmentOne"/>
  </annotation> -->
</reaction>
<reaction id="Reaction5" reversible="false">
  <listOfReactants>
    <speciesReference species="F"/>
    <speciesReference species="G"/>
  </listOfReactants>
  <listOfProducts>
    <speciesReference species="H"/>
  </listOfProducts>
  <kineticLaw>
    <math xmlns:MesoRD="http://www.w3.org/1998/Math/MathML">
    <apply>
      <times/>
      <ci>k5</ci>
      <ci>F</ci>
      <ci>G</ci>
    </apply>
  </math>
  </kineticLaw>
 <!-- <annotation xmlns:MesoRD="http://www.icm.uu.se">
    <MesoRD:location MesoRD:compartment="CompartmentOne"/>
  </annotation> -->
</reaction>
<reaction id="Reaction6" reversible="false">
  <listOfReactants>
    <speciesReference species="H"/>
  </listOfReactants>
  <listOfProducts>
    <speciesReference species="F"/>
    <speciesReference species="G"/>
  </listOfProducts>
  <kineticLaw>
    <math xmlns:MesoRD="http://www.w3.org/1998/Math/MathML">
    <apply>
      <times/>
      <ci>k6</ci>
      <ci>H</ci>
    </apply>
  </math>
  </kineticLaw>
 <!-- <annotation xmlns:MesoRD="http://www.icm.uu.se">
    <MesoRD:location MesoRD:compartment="CompartmentOne"/>
  </annotation> -->
</reaction>
<reaction id="Reaction7" reversible="false">
  <listOfReactants>
    <speciesReference species="H"/>
    <speciesReference species="I"/>
  </listOfReactants>
  <listOfProducts>
    <speciesReference species="J"/>
  </listOfProducts>
  <kineticLaw>
    <math xmlns:MesoRD="http://www.w3.org/1998/Math/MathML">
    <apply>
      <times/>
      <ci>k7</ci>
```

```
         <ci>H</ci>
         <ci>I</ci>
       </apply>
     </math>
      </kineticLaw>
     <!-- <annotation xmlns:MesoRD="http://www.icm.uu.se">
        <MesoRD:location MesoRD:compartment="CompartmentOne"/>
      </annotation> -->
    </reaction>
    <reaction id="Reaction8" reversible="false">
      <listOfReactants>
        <speciesReference species="J"/>
      </listOfReactants>
      <listOfProducts>
        <speciesReference species="A"/>
      </listOfProducts>
      <kineticLaw>
        <math xmlns:MesoRD="http://www.w3.org/1998/Math/MathML">
       <apply>
         <times/>
         <ci>k8</ci>
         <ci>J</ci>
       </apply>
     </math>
      </kineticLaw>
     <!-- <annotation xmlns:MesoRD="http://www.icm.uu.se">
        <MesoRD:location MesoRD:compartment="CompartmentOne"/>
      </annotation> -->
    </reaction>
  </listOfReactions>


</model>
</sbml>
```
