# Supplementary figures and images for: STEPS: efficient simulation of stochastic reaction–diffusion models in realistic morphologies
Source: BMC Syst Biol. 2012 May 10;6:36. doi: 10.1186/1752-0509-6-36 (PMC3472240; doi:10.1186/1752-0509-6-36)

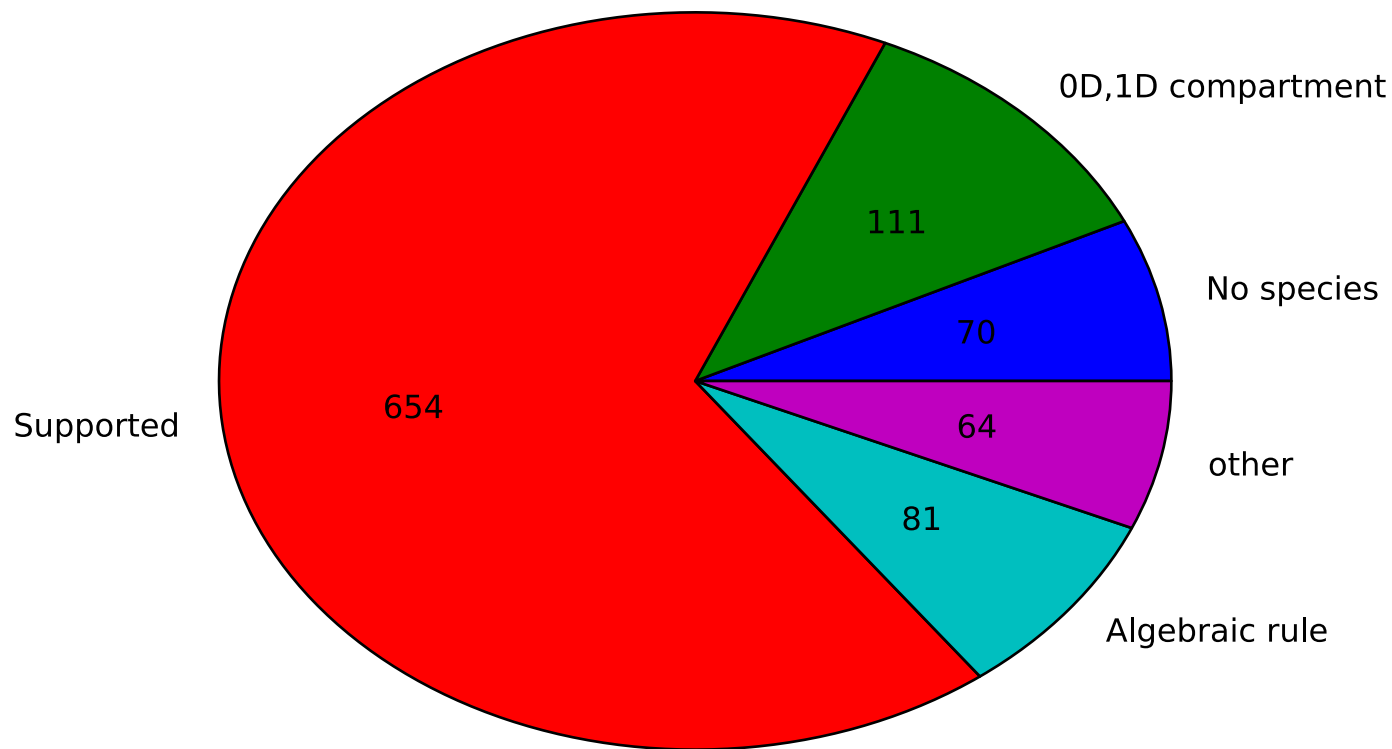

Supplement: Additional file 6 — Figure S1. SBML Test Suite support. All 980 models (l3v1) of the SBML Test Suite 2.0.0 (as of 2011/06/01) were imported, run in the Wmrk4 deterministic solver in STEPS and results compared against given solutions. The chart shows the proportion of models supported (red) and unsupported (other colors). The unsupported models are separated into 4 categories. [file 1752-0509-6-36-S6.pdf]
